# Synthesis and Pharmacological Evaluation of 6,7-Dihydro‑3*H*‑Oxazolo[3,4‑*a*]Pyrazine-5,8-Dione Compounds as Inhibitors of Phosphodiesterases 4 and 5

**DOI:** 10.1021/acsmedchemlett.5c00525

**Published:** 2026-01-20

**Authors:** Débora Rocha Helfstein, Marcio Fernando das Virgens, Julio Alejandro Rojas Moscoso, Tiago Zaminelli, Fabiano Travanca. Toledo, Jeniffer Maia de Lima, Larissa Ozols Medeiros, Bianca Alves Marcello, Vinícius Marques Soares, Gabriela Reolon Passos, Sarah Saraiva de Padua, Matheus Eduardo Gonçalves Wolf, Leonardo Martins Carneiro, Gilberto De Nucci, Artur Franz Keppler, Fabíola Zakia Mónica

**Affiliations:** † Department of Pharmacology, Faculty of Medical Sciences, 28132 University of Campinas, Sao Paulo, 13083-872, Brazil; ‡ Biolab Sanus Pharmaceutical, São Paulo, 04538-133, Brazil; § Centro de Ciências Naturais e Humanas, 74362 Universidade Federal do ABC, Avenida dos Estados, 5001, Bloco A, Santo André, São Paulo, 09210-580, Brazil

**Keywords:** Phosphodiesterases, Cyclic Nucleotides, Benign Prostatic Hyperplasia, Pictet-Spengler reaction, Oxazol, Tryptophan, dihydro[1,3]oxazolo[3,4-*a*]piperazine-5,8-dione derivatives, Docking, molecular modeling

## Abstract

Benign prostatic hyperplasia (BPH) is a prevalent condition in aging men that negatively affects the quality of life. Current therapeutic strategies aim to reduce prostate size and smooth muscle contraction. In this study, five novel 4-oxazoline-centered compounds with potential phosphodiesterase (PDE) inhibitory activity were synthesized and tested for biochemical and pharmacological effects in isolated prostate cells and tissues. Among them, compound VIII exhibited inhibitory activity against PDE5 and two PDE4 isoforms in cell-free assays. Additionally, it relaxed isolated rat prostate tissue, enhanced nitric oxide-induced relaxation, and reduced contractile responses mediated by alpha-1 adrenoceptors. Moreover, compound VIII significantly inhibited the proliferation of a human hyperplastic prostate cell line, mimicking the effects of rolipram, a PDE4 inhibitor. With its dual inhibition of PDE4 and PDE5 and its ability to decrease both contraction and cell proliferation, compound VIII emerges as a promising candidate for further investigation as a potential treatment for BPH. Complementary molecular docking and conformational analyses provided a structural rationale for the observed activity trends, highlighting the role of hydroxyethyl substituent interactions with the PDE5A H-loop in modulating inhibitory potency. Taken together, the dual PDE4/PDE5 inhibition profile of compound VIII, combined with its favorable functional effects on smooth muscle tone and cell proliferation, identifies this scaffold as a promising starting point for further optimization toward BPH therapy.

## Graphical abstract

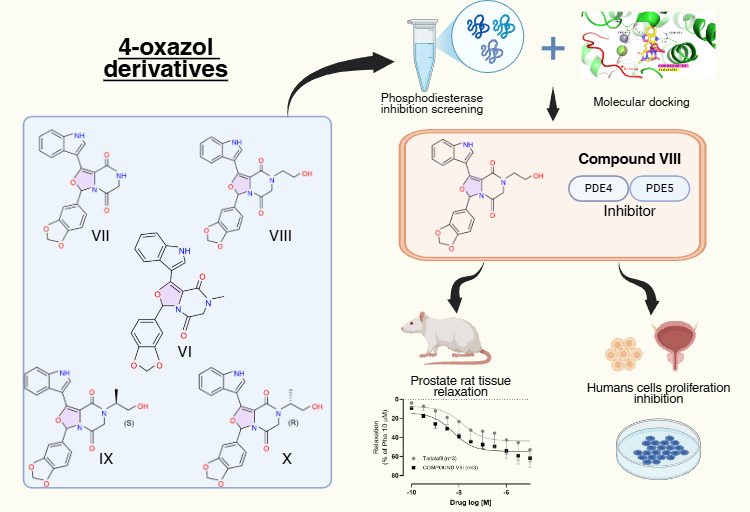

## Introduction

Cyclic nucleotide phosphodiesterases (PDEs) play a critical role in regulating the intracellular levels of cyclic adenosine monophosphate (cAMP) and cyclic guanosine monophosphate (cGMP). PDEs are a diverse group of enzymes, with multiple isoforms classified into 11 distinct families (PDE1–PDE11).^1^ Among them, PDEs from subfamilies 4, 7, and 8 hydrolyze preferentially cAMP, while PDEs from subfamilies 5, 6, and 9 hydrolyze preferentially cGMP. Additionally, PDEs from subfamilies 1, 2, 3, 10, and 11 can hydrolyze both cyclic nucleotides.^1^

The modulation of second messenger concentrations plays a key role in various physiological processes, including vascular and nonvascular smooth muscle relaxation,^2^ platelet aggregation,^2^ immune function,^3,4^ heart rhythms,^5^ and lung function,^6,7^ among others. PDE enzymes are widely distributed across different tissues in the body. As a result, inhibiting specific PDE isoforms has become an important therapeutic strategy. For instance, PDE inhibitors are approved in the clinical settings to the treatment of chronic obstructive pulmonary disease (COPD, PDE4 inhibitor),^8^ peripheral arterial disease^9^ (PDE3 inhibitor), heart failure^10^ (PDE3 inhibitor), pulmonary hypertension^7^ (PDE5 inhibitor), erectile dysfunction^11^ (PDE5 inhibitor), and benign prostatic hyperplasia^12,13^ (PDE5 inhibitor).

Benign prostatic hyperplasia (BPH) is a noncancerous condition characterized by the enlargement of the prostate. Several factors, including aging, obesity, diabetes, and metabolic syndrome, contribute to an increased incidence of BPH.^14^ Currently, the main pharmacological treatments for BPH include alpha-1 adrenoceptor antagonists, tadalafil, and 5-alpha-reductase inhibitors.^14,15^ However, despite advancements in treatment options, there are still notable drawbacks, such as side effects (particularly with alpha-1 adrenoceptor antagonists and 5-alpha-reductase inhibitors), delayed onset of relief, and surgical risks.^16^ Tadalafil is the only PDE5 inhibitor approved for the clinical treatment of BPH, particularly for patients who also have erectile dysfunction.^12^ In stromal cells isolated from hyperplastic prostate tissue, an increase in PDE5 protein expression was observed in patients with early stage BPH (those under 50 years old) compared with older patients (over 50 years old) or healthy controls. Adding tadalafil (600 nM) was found to suppress autophagy and reduce fibroblast activation.^17^ Overall, tadalafil has been shown to be noninferior to alpha-adrenoceptor blockers in improving symptoms and quality of life in BPH patients.^18^ The role of PDE4 has been extensively studied in the context of prostate cancer. For example, lower expression of the PDE4D7 isoform has been associated with poor prostate cancer outcomes^19,20^ and can be used as a possible biomarker to predict outcome.^21,22^ Additionally, roflumilast, the only PDE4 inhibitor approved in the clinical settings to the treatment of COPD, has been shown to reduce cisplatin toxicity in rat testes while enhancing its anticancer effects in prostate cancer cell lines.^23^ To date, no selective inhibitors have been developed to target a specific PDE4 isoform. According to The Human Protein Atlas, the PDE4D isoform exhibits the highest protein levels in smooth muscle and glandular prostate cells compared to PDE4B. No protein levels were detected for the isoforms PDE4A and PDE4C (source: The Human Protein Atlas). In this study, we describe the synthesis and pharmacological characterization of a novel class of 4-oxazolines. These compounds demonstrate inhibitory activity against PDE4 isoforms, as well as PDE5, and we evaluated their efficacy in isolated rat prostate tissues and human prostate cell lines.

## Results and Discussion

### Chemical Synthesis

The synthetic route to obtain the oxazoline derivatives began with the oxidation of compound **I** using DDQ. After several unsuccessful attempts to oxidize the benzylic side chain with various oxidants (such as Oxone, IBX, and SeO2),^24^ the protected compound **I** was successfully oxidized with DDQ,^25^ yielding compound **II** of interest (Scheme 1), a white crystalline solid.

**Scheme 1:**
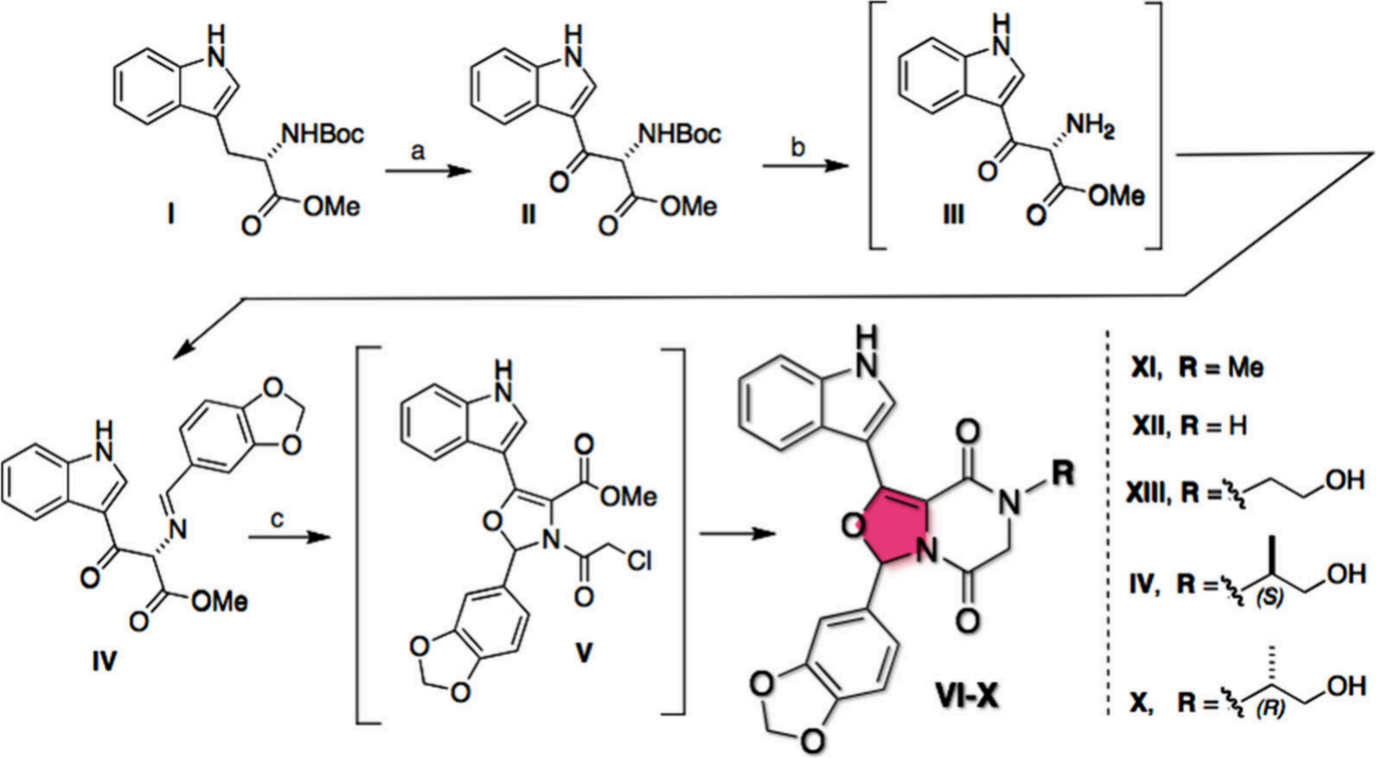
Synthesis of Compounds VI–X^a^

a Reagents and conditions: a) DDQ, THF:H_2_O (9:1), 2h, r.t.; b) HCl, MeOH, reflux; then NaHCO_3_, piperonaldehyde (2 steps, one-pot);^26^ c) chloroacetyl chloride, dry THF, piridine, 6h, r.t., then primary amines, EtOH, 16h, r.t. (2 steps, one pot).

In the subsequent reaction, the imine (**IV**) was synthesized in two steps.^26^ First, the amino group was deprotected *in situ* upon treatment with concentrated HCl in methanol. In the second step, the amine (**III**) was reacted with piperonaldehyde under appropriate pH conditions to form imine (**IV**). After filtration, a crystalline solid was obtained in excellent yield.

In the next step in the synthesis of new oxazole derivatives, the formation of the oxazoline group (Scheme 1) begins with the acylation of the imine group with chloroacetyl chloride, followed by a cyclization reaction. This results in the formation *in situ* of intermediate **V**, which is not isolated.

In this key step, the asymmetric center of the L-tryptophan precursor is naturally lost during the formation of compound **V**, leading to the formation of a double bond (4-oxazoline) and the generation of a new stereogenic center, all occurring in the same step (Scheme 1).

Adding methylamine to the intermediate **V** and stirring the solution at room temperature for 16 h led to the formation of compound **VI**. After this period, THF was removed under vacuum, and the solvent was exchanged for ethanol, which precipitated the product **VI**.^26^ The compound was then isolated by filtration as a yellowish-crystalline solid in 34% yield.

Following the same reaction pathway described in Scheme 1, a series of novel 4-oxazoline derivatives were synthesized using different primary amines (Scheme 1). Treatment with NH_4_OH led to the formation of product **VII** in 28%^26^ yield. Similarly, the use of ethanolamine afforded product **VIII** in 45%^27^ yield. Compound **IX** was obtained by reacting intermediate **V** with l-alaninol, resulting in a 21%^27^ yield. In a comparable manner, reaction of intermediate **V** with d-alaninol yielded product **X** in 25%.^27^

#### Phosphodiesterase Assay

Initially, the compounds were tested in an *in vitro* enzymatic assay (BPS Biosciences, San Diego, CA) to assess their inhibitory activity against human PDE4A1A, PDE4B1, PDE4B2, PDE4C1, PDE4D2, PDE4D3, and PDE5 enzymes. The percentage of inhibition at 1 μM was determined in triplicate. Compounds exhibiting >50% inhibition were considered to represent significant inhibition. The results are presented in Table 1.

**Table 1:** Determination of the Inhibitory Effect of Compounds VI, VII, VIII, IX and X against Different Subtypes of Phosphodiesterases

Compounds	PDE4A1A	PDE4B1	PDE4B2	PDE4C1	PDE4D2	PDE4D3	PDE5
**VI**	6.0%	27.9%	0.0%	0.0%	29.0%	3.2%	110.0%
**VII**	4.0%	18.4%	0.0%	9.0%	15.0%	73.0%	89.6%
**VIII**	–2.1%	0.0%	60.0%	0.0%	55.0%	0.0%	91.0%
**IX**	2.0%	39.8%	28.0%	0.0%	6.0%	28.0%	68.0%
**X**	7.0%	24.1%	96.0%	25.0%	9.0%	21.0%	18.0%

Additionally, Compound VIII—the compound that inhibited more enzyme subtypes than any other—was further evaluated against additional PDE families. In addition to inhibiting PDE5, PDE4B2, and PDE4D2, Compound VIII also showed significant inhibition of PDE6 and PDE11, with inhibition levels exceeding 50%. The experiments were carried out at Eurofins Cerep, Poitiers, France (Table 2).

**Table 2:** Compound VIII PDE Profile^a^

	% Inhibition of control values		
PDE subtype	first	second	Mean
**PDE1B**	–6.1	–4.6	–5.4
**PDE2A**_**1**_	–1.0	0.1	–0.4
**PDE3A**	–0.1	–2.3	–1.2
**PDE3B**	–3.7	3.3	–0.2
**PDE4A**_**1A**_	–2.9	–1.3	–2.1
**PDE4B**_**1**_	16.8	–4.2	11.9
**PDE4D**_**2**_	56.0	49.1	52.5
**PDE5**	91.3	90.7	91.0
**PDE6**	84.9	86.7	85.8
**PDE7A**	0.5	0.1	0.3
**PDE8A**_**1**_	0.0	–3.1	–1.5
**PDE10A**_**2**_	26.8	42.6	34.7
**PDE9A**_**2**_	1.0	1.0	1.0
**PDE11A**_**4**_	72.2	75.9	74.1

a Results are shown as% inhibition at concentration 10 μM, in duplicate.

By modifying the **R** group, we also alter the molecule’s specificity toward the tested enzyme. For instance, when d-alaninol was used, **Compound X** lost its specificity for PDE5. In contrast, **Compound VIII** not only showed strong inhibitory potential against PDE5 but also inhibited two isoforms of the PDE4 family. This dual inhibition may offer therapeutic benefits for treating BPH.^28–31^

#### Computational Studies

Molecular modeling simulations were employed to examine the binding modes of the novel 4-oxazoline-centered compounds within the catalytic site of PDE5A, using the crystallographic structure of the PDE5A–tadalafil complex (PDB-ID: 1UDU) as the search model. This structure was selected because it contains the reference scaffold inhibitor tadalafil in a cocrystallized binding pose and corresponds to a nonchimeric PDE5A crystal structure, in contrast to other available entries such as 1XOZ,^32^ thereby offering a structurally relevant framework for the comparative evaluation of binding modes of the newly designed compounds. Docking calculations indicate that compounds **VI–X** adopt closely related binding poses and generate predicted binding affinities (ΔG) comparable to the value calculated for the reference compound tadalafil (Figure S1 in the Supporting Information). Notably, the hydrogen bond formed between the indole moiety of **VI–X** and TYR612 occurs in a position opposite to the corresponding interaction observed between tadalafil and GLN817 (Figure 1). It has been extensively documented that GLN817 is a key residue involved in cGMP/cAMP specificity control^33^ and is known to establish interactions with different classes of PDE5 inhibitors.^34,35^

**Figure 1:**
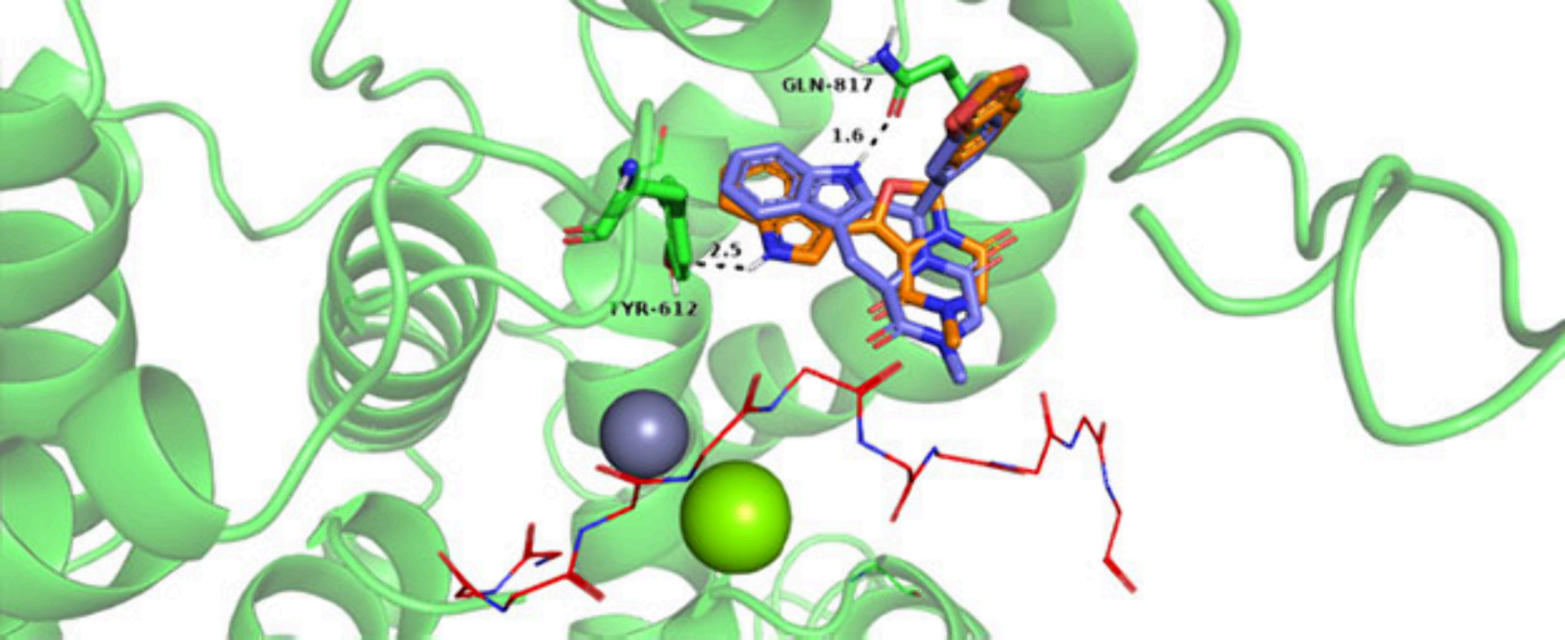
Superposed binding modes of tadalafil and compound VI at the PDE5A catalytic site, as predicted by molecular docking. H-bonding interactions are indicated by black dashed lines, with interatomic distances labeled in Å, highlighting the interactions between tadalafil (blue sticks) and GLN817 and between Compound VI (orange sticks) and TYR612. Additional ligand–protein interactions are presented in the corresponding 2D interaction map (Figure S2 in the Supporting Information). The H-loop backbone is shown as a red ribbon. Docking poses were aligned in PyMOL to the reference X-ray crystallographic structure (PDB ID: 1UDU) to enable the placement of the catalytic metal ions Zn^2+^ (gray sphere) and Mg^2+^ (green sphere), facilitating visualization of the binding mode and comparison with previously reported PDE5A–ligand complexes.

The hydrophobic interactions were established by compounds **VI-X** with Phe820, Leu804, Phe786, Val782, Ile768, and Ala767 (Figure S2) and did not allow us to derive a coherent structure–activity relationship capable of explaining the PDE5A inhibition assay results (Table 1). To gain further insight, we focused our analysis on compounds **VIII**, **IX**, and **X**, which exhibit markedly different PDE5A inhibitory activities despite sharing a structurally similar substituent attached to the piperazinedione ring. Figure 2 shows that their terminal hydroxyl groups are oriented toward the H-loop. Compounds **VIII** and **IX** establish H-bond interactions with the H-loop backbone, whereas compound **X** preferentially forms an intramolecular H-bond with its piperazinedione carbonyl group.

**Figure 2:**
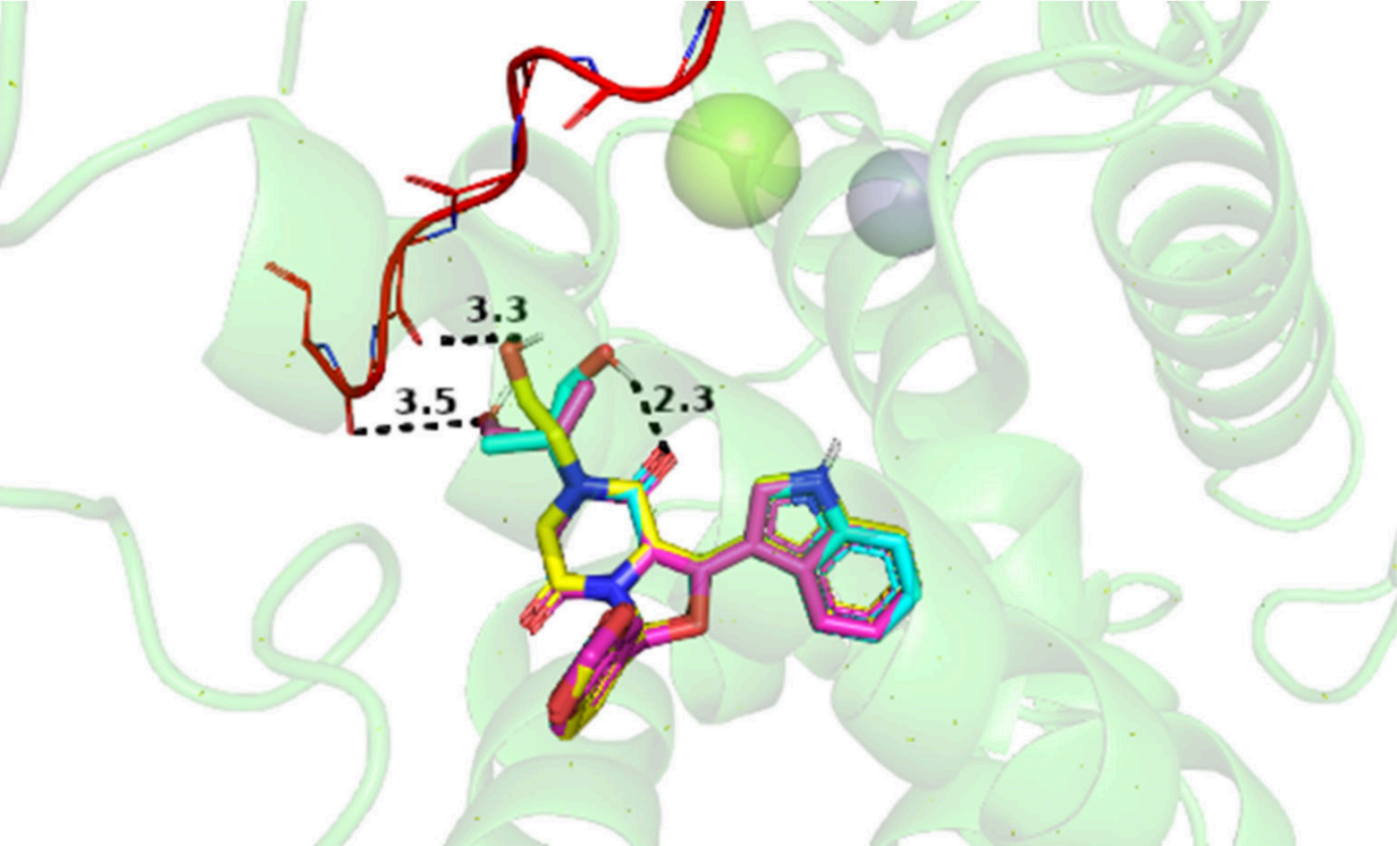
Close-up view of compounds VIII (magenta), IX (yellow), and X (light blue) positioned near the backbone of the PDE5A H-loop (red ribbon), highlighting the intramolecular H-bond formed by Compound X with its piperazinedione carbonyl group. Dashed black lines denote H-bonding interactions with interatomic distances labeled in Å. The PyMOL program was used to process the molecular docking outputs and generate images, rendering enzyme cartoon representations and metallic ions at 80% transparency to facilitate inspection of the ligand poses and H-bond interactions.

To clarify the relationship between the distinct binding orientations of the hydroxyethyl substituents and the inhibitory activities measured for Compounds **VIII–X**, rotation about the N7–C2 σ bond (Figure 3A) was first evaluated *in vacuo* as an isolated system. The DFT-calculated rotamer energy profile, displaying a maximum energy difference of only 3.72 kcal·mol^–1^ (Figure S3 in the Supporting Information), indicates that intrinsic steric effects along the N7–C2 σ bond are not sufficient, on their own, to account for the conformational preferences observed in the binding site. Inspection of the PDE5A crystal structure reveals that the hydroxyethyl substituents of **VIII**, **IX**, and **X** occupy a well-defined hydrophobic cleft formed by LEU840, PHE186, and VAL782 on the lower face and ALA726, LEU725, and ILE824 on the upper face (yellow and blue surfaces, respectively) (Figure 3B and Figure S4 in Supporting Information). This spatially confined environment imposes pronounced steric constraints that limit rotation about the N7–C2 σ bond. In agreement with this structural context, Newman projections (Figure 3B) illustrate the conformational space accessible to the C2 substituents. At this constrained subpocket architecture, access to local N7–C2 σ bond rotamer energy minima—represented by conformers **1** and **4**—requires appropriate orientation of the C2 hydrogen toward the region highlighted in yellow, which demarcates the limits of the dihedral space associated with the highest steric repulsion.

**Figure 3:**
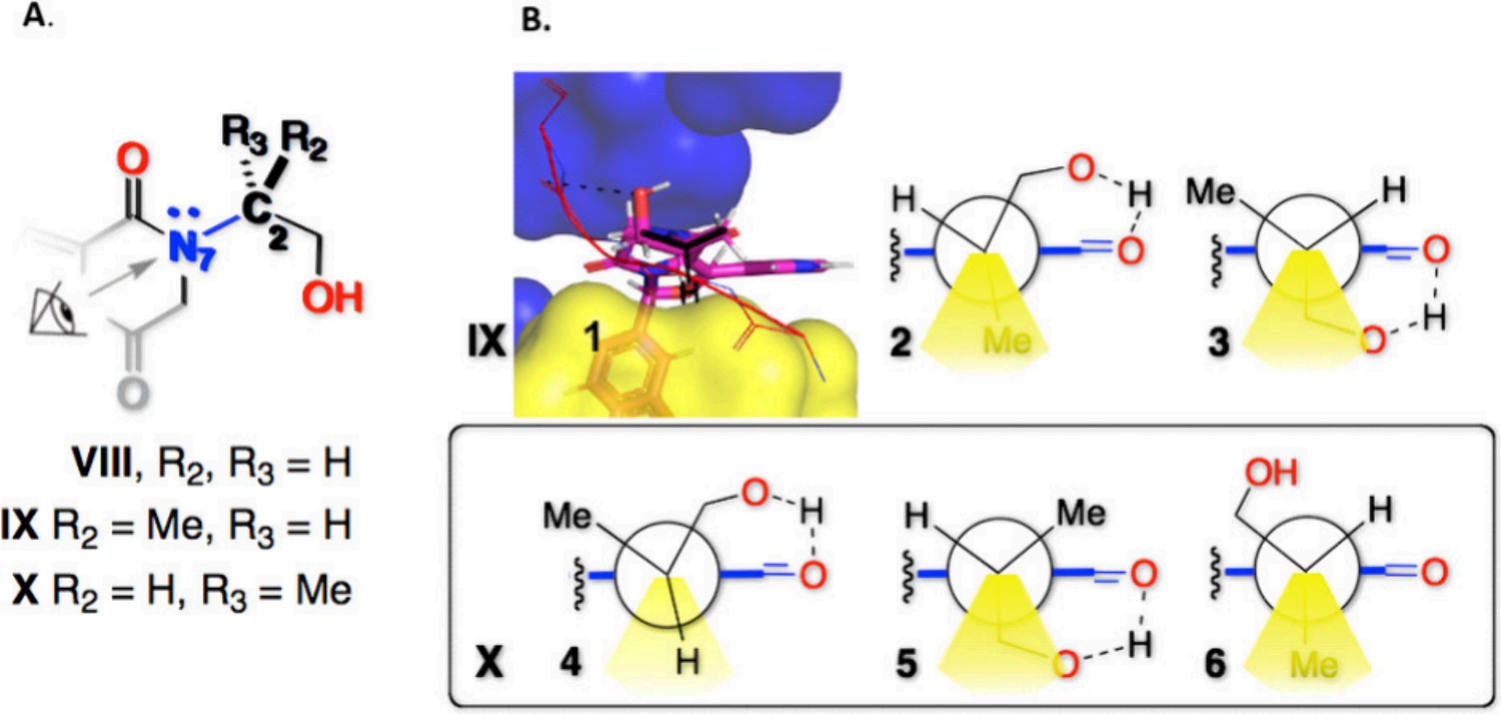
A. Chemical structures of compounds VIII, IX, and X, focusing on the N7–C2 sp^2^–sp^3^ σ bond. B. Newman projections of compounds IX and X illustrating the rotation about the N7–C2 sp^2^–sp^3^ σ bond. The N7 atom adopts a quasiplanar geometry as a result of conjugation between its lone pair and the adjacent carbonyl π system, which influences the conformational preferences along this bond. The dihedral space with the greater steric hindrance is highlighted in green. The eye, the H-loop, and the green spots were inserted into the chemical structures using the GNU Image Manipulation Program (GIMP).

To experimentally validate these conformational restrictions, redocking experiments were performed using the top-ranked poses of **IX** and **X** (Conformers 1 and 4). Controlled rotation of the N7–C2 σ bond was used to generate alternative input geometries (Conformers 2 and 6). Upon redocking, these structures consistently relaxed back to Conformers 1 and 4 (Figure S5 in Supporting Information), confirming that the steric environment of the hydroxyethyl-accommodating subpocket dictates the local binding geometry by filtering out alternative rotamers.

These findings suggest that when locked in their local conformational energy minima, the hydroxyl group of **IX** (Conformer 1) is optimally oriented for stabilizing interactions within the H-loop. Conversely, in **X** (Conformer 4), the hydroxyl group is distal to the H-loop, favoring an intramolecular hydrogen bond that probably alleviates oxygen lone pair electronic repulsion.

The superior inhibitory activity of **VIII** relative to **IX** and **X** is attributed to the lack of C2 methyl substituents; the two hydrogen atoms reduce the steric bulk, granting the flexibility necessary to sample multiple interaction sites along the flexible H-loop. Because compounds **VI** and **VII** possess different N7 substituents, they may target distinct regions of the PDE5A pocket. These hypotheses and a more detailed binding behavior of **VIII–X** are currently being investigated via molecular dynamics simulations.

##### *In Vitro* Functional Assays

Next we carried out concentration–response curves to Compound VIII in prostates from rats precontracted to the alpha-1 adrenoceptor agonist to determine the potency (pEC50) and maximal response (Emax) values. As demonstrated in Figure 4, the pharmacological parameters of Compound VIII did not differ significantly in comparison with tadalafil, a PDE5 inhibitor (Figure 4A-C), thus showing that Compound VIII presents similar potency and efficacy of that of tadalafil. In this protocol, tadalafil was used as a comparator, as it is the only PDE inhibitor approved in the treatment of patients with BPH.^12^

**Figure 4:**
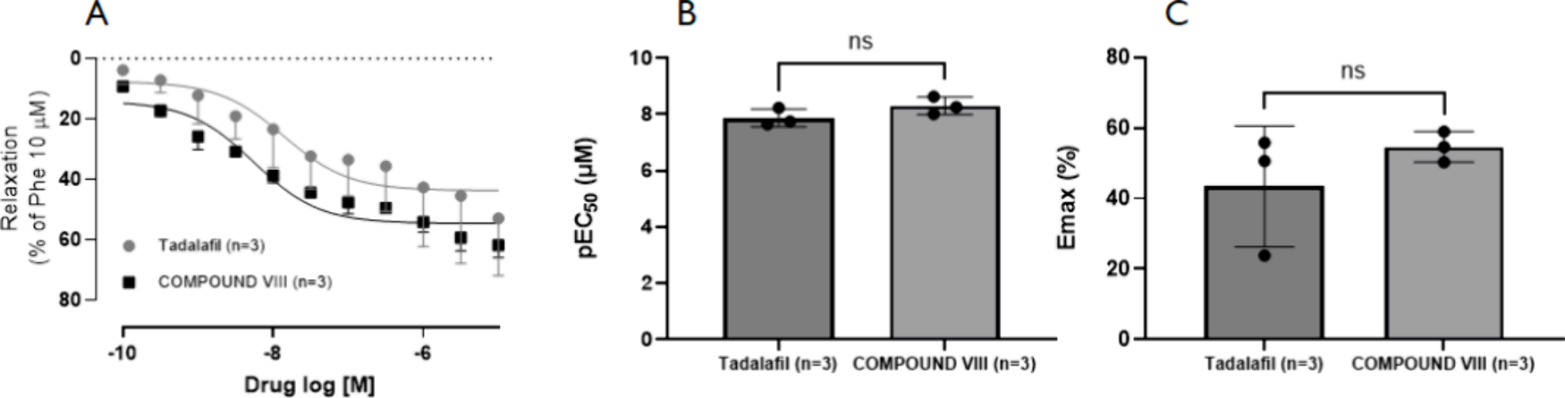
Relaxation response to Compound VIII and Tadalafil in isolated rat prostate precontracted with PE (10 uM), which was taken as 100%. Concentration–response curves to Compound VIII and tadalafil. Values have been presented as Mean ± SD (Student’s *t* test); *n* = 3.

Two additional functional assays were conducted with rat prostate strips in both the presence and absence of **Compound VIII**. The first assay involved cumulative addition (100 pM to 100 μM) of sodium nitroprusside (SNP), a NO donor, while the second involved cumulative addition of isoproterenol (a nonselective β-adrenergic receptor agonist). Since **Compound VIII** inhibited PDE4 and PDE5, the purpose of this experiment was to determine whether this compound could enhance the relaxations induced by substances that increase cGMP and cAMP levels. In the SNP assay (Figure 5A-C), **Compound VIII** significantly potentiated SNP-induced relaxation. In the isoproterenol assay (Figure 5D-F), **Compound VIII** did not potentiate isoproterenol-induced relaxation. In another experiment, we also carried out a concentration–response curve to isoproterenol in the presence of another PDE4 inhibitor, roflumilast (100 nM, Figure 5G-I). Surprisingly, even roflumilast did not potentiate the relaxation induced by isoproterenol. This suggests that, at least in the smooth muscle reactivity, Compound VIII favors the relaxation induced by the nitric-oxide donor, SNP.

**Figure 5:**
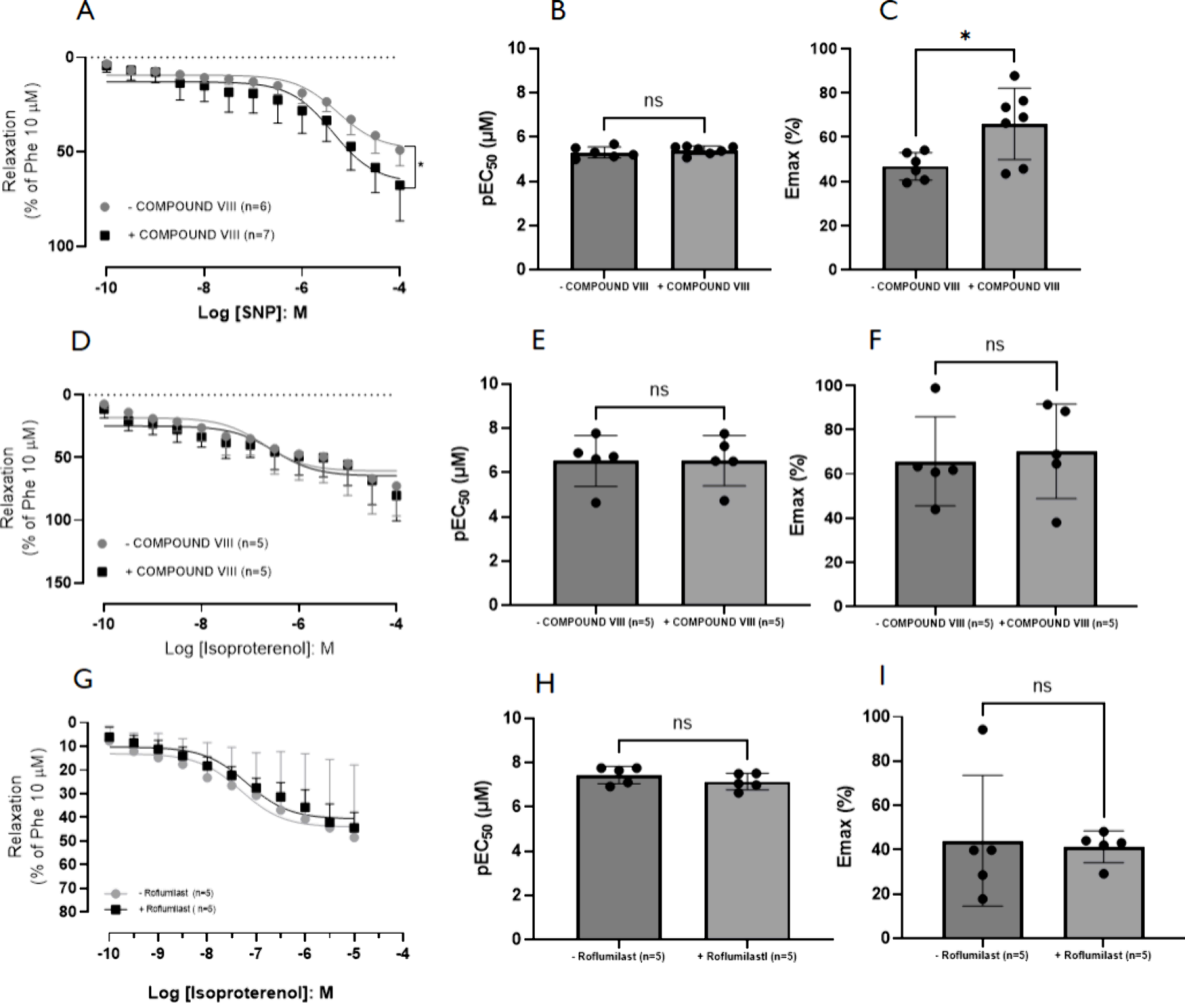
Relaxation results in prostatic tissue from rat, in the absence (*n* = 6) or presence (*n* = 7) of Compound VIII (3 μM) in tissues precontracted with phenylephrine, which was taken as 100%. **(A-C)** Concentration–response curves to the nitric oxide donor sodium nitroprusside (SNP). **(D-I)**. Concentration–response curves to the beta-adrenoceptor agonist isoproterenol in the absence (D-F) and presence (G-I) of roflumilast (100 nM). Values are presented as mean ± SD (Student’s *t* test).

The final assay evaluated the contraction response of prostate tissue to phenylephrine (PE) with and without **Compound VIII**. As shown in Figure 6, **Compound VIII** reduced the contraction potency of PE, shifting the curve 7-fold to the right. The **EC50** in the presence of **Compound VIII** was (3.93 ± 0.18) × 10^–6^ μM, compared to (5.39 ± 0.11) × 10^–7^ μM in its absence (*p* < **0.05**; **n** = **5**). This further supports the potential of **Compound VIII** as an effective smooth muscle relaxant.

**Figure 6:**
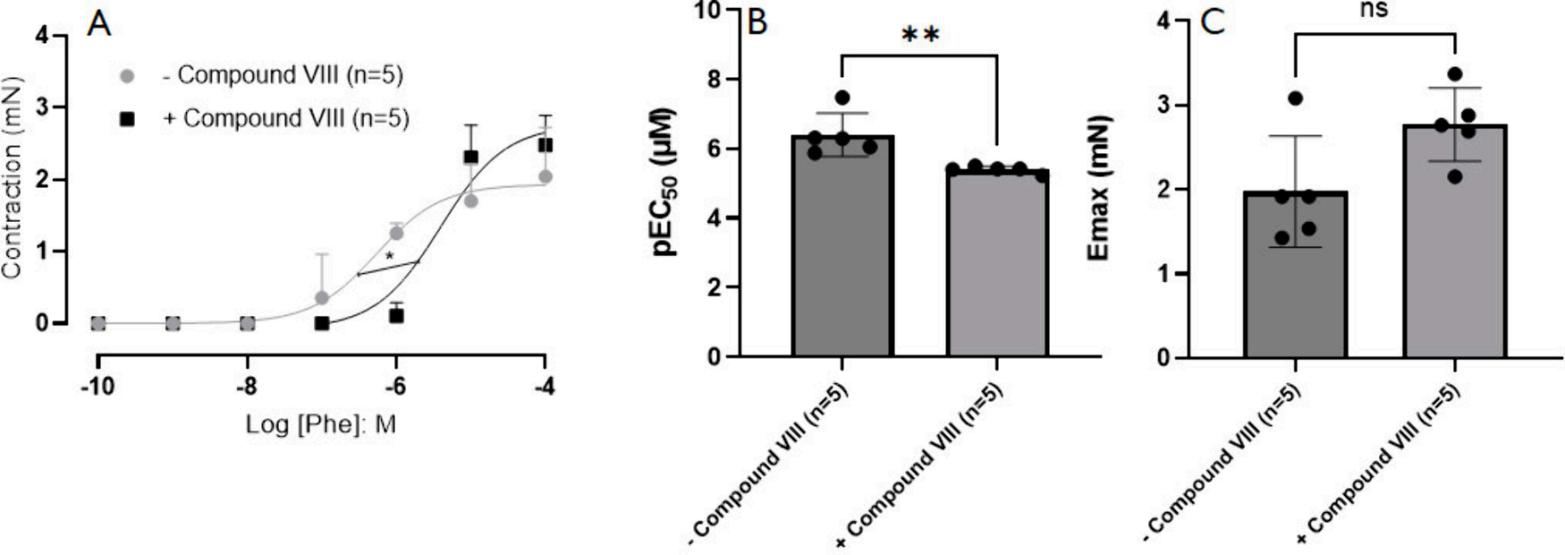
Concentration–response curves to the alpha-1 adrenoceptor receptor agonist, phenylephrine, in prostatic tissue from rat in the absence (*n* = 6) or presence (*n* = 7) of Compound VIII (3 μM) in tissues contracted with phenylephrine. Values are presented as mean ± SD (Student’s *t* test).

The results of the functional tests show that Compound VIII induces the relaxation of prostatic smooth muscle. This relaxation is likely due to the inhibition of PDE5—an isoform presents in both human and rat prostates^29,30,36^ — as previously observed with tadalafil and other PDE5 inhibitors.^37,38^ Compound VIII potentiated the relaxation induced by the NO donors. Although nonselective PDE4 inhibitors relax prostatic smooth muscle^39^ we did not observe any potentiation in the relaxation induced by the beta-adrenoceptor agonist, isoproterenol, when Compound VIII or the positive control (roflumilast) was incubated. Compound VIII also inhibits PDE4, specifically PDE4B and PDE4D,^29,30^ both of which are found in the human prostate. While previous studies have shown that PDE4 inhibitors relax prostatic smooth muscle, our compound primarily promotes relaxation through cGMP-augmenting substances rather than through beta-adrenoceptor agonists. This suggests the possibility of cAMP compartmentalization, and it may indicate that the beta-adrenoceptor protein is not in close proximity to PDE4.^39^

### Cells Proliferation Assays

Zenzmaier et al.^40^ and Powers et al.^41^ have demonstrated that PDE5 and PDE4 inhibitors attenuated human prostatic cells proliferation, so we decided to perform the cell proliferation assay with immortalized human prostatic stromal myofibroblast cell line (WPMY-1), human prostate epithelial cells (RWPE-1), and human benign prostatic hyperplasia cell line (BPH-1). The results demonstrated that Compound VIII (0.003–3 μM) significantly reduced cell proliferation in all three cells lines tested, as shown in Figure 7(A-C), with higher magnitude of inhibition in the stromal cells (WPMY-1).

**Figure 7:**
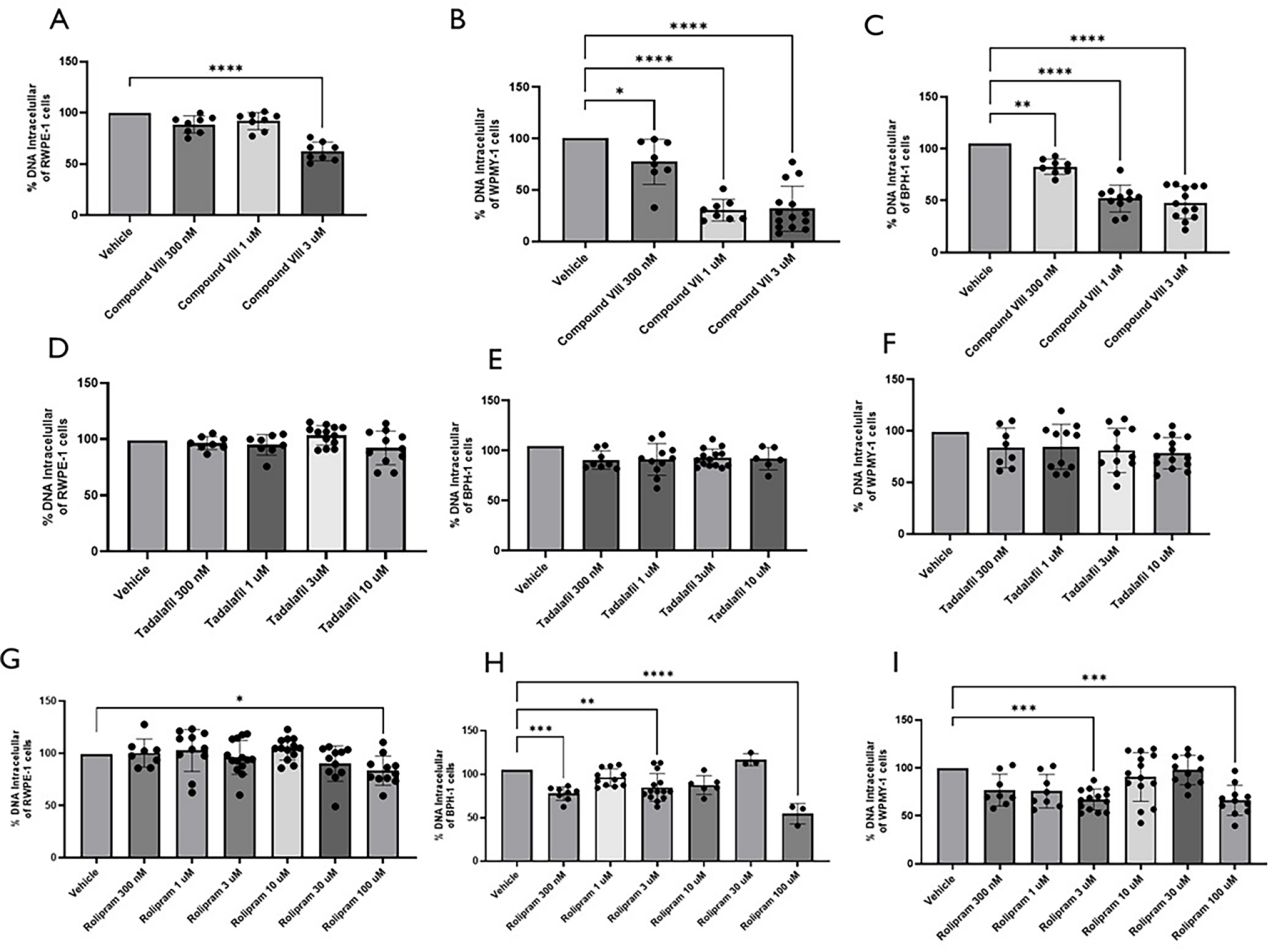
**Cell Proliferation Inhibition Assay (%) with RWPE-1, BPH-1, and WPMY-1 cells lines:** (A-C) Results from Compound VIII; (D-F) Results from Tadalafil; (G-I) Results from Rolipram. Cell proliferation was assessed using CyQUANT assay kits for all cell lines. Data are presented as the mean ± SD from three to four independent experiments, each performed in triplicate. Comparisons were made between the vehicle (DMSO 0.1%) and treatment groups. Statistical analysis was conducted using one-way ANOVA, followed by Bonferroni’s multiple comparisons test. *p < 0.05; ** *p* < 0.01; *** *p* < 0.001; **** *p* < 0.0001 compared to the vehicle group.

We also performed the same assay using two other phosphodiesterase inhibitors: tadalafil (0.003 – 10 μM), a PDE5 inhibitor (Figure 7D-F), and rolipram (0.003 – 100 μM), a PDE4 inhibitor (Figure 7G-I). Tadalafil exhibited modest antiproliferative effects in the BPH-1 cell line but only at the highest concentration tested. In contrast, rolipram inhibited cell proliferation across all three cell lines, with the most pronounced effect observed in the hyperplastic cell line. In the other two cell lines, PDE4 inhibition was effective, mainly at higher concentrations. Due to its low solubility, we were unable to test Compound VIII and tadalafil at higher concentrations as this interfered with the cell assays.

The antiproliferative effects observed with the new compound Compound VIII may be explained, in part, by the synergistic inhibition of PDE4 and PDE5, as these isoenzymes can be expressed and upregulated in human prostatic stromal and hyperplastic tissue.^29–31,42,43^ This inhibition leads to elevated levels of cAMP and cGMP, respectively, in the cells. There is evidence suggesting that prostatic cell proliferation can be mediated through the PKA^41^ and PKG^40^ pathways, which are activated by these second messengers, cAMP and cGMP. Another important factor to consider is the inhibition of the PDE4D isoenzymes, which have previously been associated with reduced growth of prostate tumor cells.^41,44^ These isoforms are also targeted by Compound VIII, and their inhibition may contribute to the decreased cell proliferation observed in the cell lines used in this study.

In summary, a novel class of 4-oxazoline compounds has been developed. Among the five synthesized molecules, Compound VIII inhibits three phosphodiesterase (PDE) families—most notably PDE4 and PDE5, which are of particular clinical relevance in the lower urinary tract.^12,45,46^ This new compound represents a promising therapeutic approach for benign prostatic hyperplasia (BPH), aligning with findings by Uckert et al.,^29^ who reported that PDE4 and PDE5 inhibitors can effectively treat lower urinary tract symptoms (LUTS) associated with BPH. Compound VIII may offer dual action: addressing the dynamic component of the disease by relieving pressure on the prostatic urethra and targeting the static component by potentially reducing the size of the enlarged prostate all within a single drug.

Safety Statement. No unexpected or unusually high safety hazards were encountered.

## Supplementary Material



## Notes

General methods for the synthesis and characterization of all compounds, methods for the enzymatic, computacional studies, functional and cellular assay data in Material, Methods and Analysis (PDF)

## Abbreviations

BPH: benign prostate hyperplasia
cAMP: cyclic Adenosine Monophosphate
cGMP: cyclic Guanosine Monophosphate
COPD: Chronic Obstructive Pulmonary Disease
DDQ: 2,3-Dichloro-5,6-dicyano-1,4-benzoquinone
IBX: 2-Iodoxybenzoic acid
LUTS: Lower Urinary Tract Symptoms
NO: nitric oxide
PDE: phosphodiesterase
PDE4: phosphodiesterase type 4
PDE4A1A: phosphodiesterase subtype 4A1A
PDE4B1: phosphodiesterase subtype 4B1
PDE4B2: phosphodiesterase subtype 4B21
PDE4C1: phosphodiesterase subtype 4C1
PDE4D2: phosphodiesterase subtype 4D2
PDE4D3: phosphodiesterase subtype 4D3
PDE5: phosphodiesterase type 5
PDE6: phosphodiesterase type 6
PDE11: phosphodiesterase type 11
PE: phenylephrine
SNP: sodium nitroprusside
THF: tetrahidrofurano

## References

[1] Surapisitchat, J. ; Beavo, J. A. Phosphodiesterase Families. In Handbook of Cell Signaling, 2/e; Elsevier Inc., 2010; Vol. 2, pp 1409–1414. 10.1016/B978-0-12-374145-5.00173-X.

[2] Francis S. H., Busch J. L., Corbin J. D. (2010). CGMP-Dependent Protein Kinases and
CGMP Phosphodiesterases in Nitric Oxide and CGMP Action. Pharmacol Rev..

[3] Serezani C. H., Ballinger M. N., Aronoff D. M., Peters-Golden M. (2008). Cyclic AMP:
Master Regulator of Innate Immune Cell Function. Am. J. Respir. Cell Mol. Biol..

[4] Azevedo L. L. d., Kummerle A. E. (2015). PDE4 Inhibitors:
From Discovery and Announced Failure
to Its Resurgence. Revista Virtual de Quimica.

[5] Lin Y., Cheng C., Huang J., Chen Y., Lu Y., Chen Y., Chen S., Chen Y. (2020). Various Subtypes of
Phosphodiesterase Inhibitors Differentially Regulate Pulmonary Vein
and Sinoatrial Node Electrical Activities. Exp
Ther Med..

[6] Page C. P. (2015). Phosphodiesterase
Inhibitors for the Treatment of Asthma and Chronic Obstructive Pulmonary
Disease. Int. Arch Allergy Immunol.

[7] Wilkins M.
R., Wharton J., Grimminger F., Ghofrani H. A. (2008). Phosphodiesterase
Inhibitors for the Treatment of Pulmonary Hypertension. Eur. Respir. J..

[8] Halpin D. M. (2008). ABCD of
the Phosphodiesterase Family: Interaction and Differential Activity
in COPD. International Journal of COPD.

[9] Robless, P. ; Mikhailidis, D. ; Stansby, G. Cilostazol for Peripheral Arterial Disease. In Cochrane Database of Systematic Reviews; John Wiley & Sons, Ltd, 2007. 10.1002/14651858.cd003748.pub2. 17253494

[10] Aiad N., du Fay de Lavallaz J., Zhang M. J., Chaikijurajai T., Ye B., Nijjar P. S., Lahiri J. A., Martin C. M., Alexy T., Meyer M. (2025). Cilostazol
in Patients with Heart Failure and Preserved Ejection
Fraction—The CLIP-HFpEF Trial. ESC Heart
Fail.

[11] Moreland R. B., Goldstein I., Kim N. N., Traish A. (1999). Sildenafil Citrate,
a Selective Phosphodiesterase Type 5 Inhibitor:: Research and Clinical
Implications in Erectile Dysfunction. Trends
in Endocrinology and Metabolism.

[12] Mónica F. Z., De Nucci G. (2019). Tadalafil for the Treatment
of Benign Prostatic Hyperplasia. Expert Opin
Pharmacother.

[13] Zahir M., Samzadeh M., Poopak A., Khoshdel A. R., Armin A. (2023). Sildenafil
Vs. Tadalafil for The Treatment of Benign Prostatic Hyperplasia: A
Single-Arm Self-Controlled Clinical Trial. Urol
J..

[14] Chughtai B., Forde J. C., Thomas D. D. M., Laor L., Hossack T., Woo H. H., Te A. E., Kaplan S. A. (2016). Benign Prostatic
Hyperplasia. Nat. Rev. Dis Primers.

[15] Madersbacher S., Sampson N., Culig Z. (2019). Pathophysiology
of Benign Prostatic
Hyperplasia and Benign Prostatic Enlargement: A Mini-Review. Gerontology.

[16] Kim E. H., Larson J. A., Andriole G. L. (2016). Management of Benign Prostatic Hyperplasia. Annu. Rev. Med..

[17] Jin, S. ; Liu, Z. ; Xiang, P. ; Fu, M. ; Zhang, G. ; Li, J. ; Niu, Y. Activation of the CGMP/PKG/ERK Signaling Pathway Associated with PDE5Is Inhibits Fibroblast Activation by Downregulating Autophagy in Early Progressive Benign Prostatic Hyperplasia. World J. Urol. 2024, 42 (1). 10.1007/s00345-024-04956-9. 38761255

[18] Casabé A., Roehrborn C. G., Da Pozzo L. F., Zepeda S., Henderson R. J., Sorsaburu S., Henneges C., Wong D. G., Viktrup L. (2014). Efficacy and
Safety of the Coadministration of Tadalafil Once Daily with Finasteride
for 6 Months in Men with Lower Urinary Tract Symptoms and Prostatic
Enlargement Secondary to Benign Prostatic Hyperplasia. Journal of Urology.

[19] Gulliver C., Busiau T., Byrne A., Findlay J. E., Hoffmann R., Baillie G. S. (2024). CAMP-Phosphodiesterase
4D7 (PDE4D7) Forms a CAMP Signalosome
Complex with DHX9 and Is Implicated in Prostate Cancer Progression. Mol. Oncol.

[20] Böttcher R., Henderson D. J. P., Dulla K., Van Strijp D., Waanders L. F., Tevz G., Lehman M. L., Merkle D., Van Leenders G. J. L. H., Baillie G. S., Jenster G., Houslay M. D., Hoffmann R. (2015). Human Phosphodiesterase 4D7 (PDE4D7)
Expression Is Increased in TMPRSS2-ERG-Positive Primary Prostate Cancer
and Independently Adds to a Reduced Risk of Post-Surgical Disease
Progression. Br. J. Cancer.

[21] Henderson D. J. P., Houslay M. D., Bangma C. H., Hoffmann R. (2019). Creating a Potential
Diagnostic for Prostate Cancer Risk Stratification (InformMDxTM) by
Translating Novel Scientific Discoveries Concerning CAMP Degrading
Phosphodiesterase-4D7 (PDE4D7). Clin Sci..

[22] Alves
de Inda M., van Strijp D., den Biezen-Timmermans E., van Brussel A., Wrobel J., van Zon H., Vos P., Baillie G. S., Tennstedt P., Schlomm T., Houslay M. D., Bangma C., Hoffmann R. (2018). Validation of Cyclic Adenosine Monophosphate
Phosphodiesterase-4D7 for Its Independent Contribution to Risk Stratification
in a Prostate Cancer Patient Cohort with Longitudinal Biological Outcomes. Eur. Urol Focus.

[23] Abdel-Wahab B. A., Walbi I. A., Albarqi H. A., Ali F. E. M., Hassanein E. H. M. (2021). Roflumilast
Protects from Cisplatin-Induced Testicular Toxicity in Male Rats and
Enhances Its Cytotoxicity in Prostate Cancer Cell Line. Role of NF-ΚB-P65,
CAMP/PKA and Nrf2/HO-1, NQO1 Signaling. Food
Chem. Toxicol..

[24] Oikawa Y., Yonemitsu O. (1976). Selective Oxidation of the Side Chain at C-3 of Indoles. J. Organic Chem..

[25] Yonemitsu O., Oikawa Y., Yoshioka T., Mohri K. (1979). Synthesis of Pimprinine
and Related Oxazolylindole Alkaloids from N-Acyl Derivatives of Tryptamine
and Tryptophan Methyl Ester by DDQ Oxidation. Heterocycles.

[26] Sacurai, S. L. ; Zaim, M. F. ; Touzarim, C. E. C. ; Keppler, A. F. ; De Nucci, G. Composto de Fórmula (I), Ou Seus Sais Farmaceuticamente Aceitáveis, Composição Farmacêutica, Uso Da Composição Farmacêutica, Uso Do Composto e Método Para Preparar o Composto. BR 112013003225-1, August 2, 2012. https://patents.google.com/patent/BR112013003225B1/pt (accessed 2025-08-30).

[27] Sacurai, S. L. ; Touzarim, C. E. C. ; Toledo, F. T. ; Sousa, B. Composto Derivado de 6,7-Dihidro-3H-Oxazolo­[3,4-A]­Pirazina-5,8-Diona, Composições Farmacêuticas, Uso Dos Compostos, Processo de Síntese Dos Compostos de Fórmula (II) e (III). BR 112016019609-0, August 24, 2015.

[28] Giuliano F., Ückert S., Maggi M., Birder L., Kissel J., Viktrup L. (2013). The Mechanism of Action of Phosphodiesterase Type 5
Inhibitors in the Treatment of Lower Urinary Tract Symptoms Related
to Benign Prostatic Hyperplasia. European Urology..

[29] Ückert S., Küthe A., Küthe K., And J., Stief C. G. (2001). Characterization
and Functional Relevance of Cyclic Nucleotide Phosphodiesterase
Isoenzymes of the Human Prostate. THE JOURNAL
OF UROLOGY.

[30] Ückert S., Oelke M., Stief C. G., Andersson K. E., Jonas U., Hedlund P. (2006). Immunohistochemical Distribution
of CAMP- and CGMP-Phosphodiesterase (PDE) Isoenzymes in the Human
Prostate. Eur. Urol.

[31] Mao Q., Li Z., Chen P., Guo Y., Xiang H., Zeng G., Xu D., Cao B., Zhao K., Xiao H., Zhang X. (2017). 391 Upregulation
of Phosphodiesterase Type 4 in the Hyperplastic Prostate. J. Sex Med..

[32] Card G. L., England B. P., Suzuki Y., Fong D., Powell B., Lee B., Luu C., Tabrizizad M., Gillette S., Ibrahim P. N., Artis D. R., Bollag G., Milburn M. V., Kim S. H., Schlessinger J., Zhang K. Y. J. (2004). Structural Basis for the Activity
of Drugs That Inhibit Phosphodiesterases. Structure.

[33] Sung B.-J., Yeon Hwang K., Ho Jeon Y., Lee J., Heo Y.-S., Moon J., Hwan Kim J., Min Yoon J., Hyun Y.-L., Kim E., Jin Eum S., Park S.-Y., Lee J.-O., Gyu Lee T., Ro S., Myung Cho J. (2003). Structure
of the Catalytic Domain of Human Phosphodiesterase 5 with Bound Drug
Molecules. Nature.

[34] Park S. Y., Pham D., Shukla P., Edward J., John R., Li A., Hadjiargyrou M., Mori M., Zuccarello E., Arancio O., Fiorito J. (2025). Discovery
of Indole-Based PDE5 Inhibitors:
Synthesis and Pharmacological Evaluation. ACS
Med. Chem. Lett..

[35] Fiorito J., Vendome J., Saeed F., Staniszewski A., Zhang H., Yan S., Deng S. X., Arancio O., Landry D. W. (2017). Identification of a Novel 1,2,3,4-Tetrahydrobenzo­[b]­[1,6]­Naphthyridine
Analogue as a Potent Phosphodiesterase 5 Inhibitor with Improved Aqueous
Solubility for the Treatment of Alzheimer’s Disease. J. Med. Chem..

[36] Wang L., Zhang X., Wang G., Visweswariah S. S., Lin G., Xin Z., Lue T. F., Lin C. S. (2015). Lobe-Specific Expression
of Phosphodiesterase 5 in Rat Prostate. Urology.

[37] Kedia G. T., Ückert S., Jonas U., Kuczyk M. A., Burchardt M. (2008). The Nitric
Oxide Pathway in the Human Prostate: Clinical Implications in Men
with Lower Urinary Tract Symptoms. World J.
Urol.

[38] Giuliano F., Ückert S., Maggi M., Birder L., Kissel J., Viktrup L. (2013). The Mechanism of Action of Phosphodiesterase
Type 5
Inhibitors in the Treatment of Lower Urinary Tract Symptoms Related
to Benign Prostatic Hyperplasia. European Urology..

[39] Kedia G. T., Ückert S., Polat H., Merseburger A. S., Kuczyk M. A. (2012). Evaluating the Significance
of Cyclic Adenosine Monophosphate-Mediated
Signaling in Human Prostate: A Functional and Biochemical Study. Urology.

[40] Zenzmaier C., Sampson N., Pernkopf D., Plas E., Untergasser G., Berger P. (2010). Attenuated Proliferation
and Trans-Differentiation
of Prostatic Stromal Cells Indicate Suitability of Phosphodiesterase
Type 5 Inhibitors for Prevention and Treatment of Benign Prostatic
Hyperplasia. Endocrinology.

[41] Powers G. L., Hammer K. D. P., Domenech M., Frantskevich K., Malinowski R. L., Bushman W., Beebe, Marker P. C. (2015). Phosphodiesterase
4D Inhibitors Limit Prostate Cancer Growth Potential. Molecular Cancer Research.

[42] Fibbi B., Morelli A., Vignozzi L., Filippi S., Chavalmane A., De Vita G., Marini M., Gacci M., Vannelli G. B., Sandner P., Maggi M. (2010). Characterization
of Phosphodiesterase
Type 5 Expression and Functional Activity in the Human Male Lower
Urinary Tract. Journal of Sexual Medicine.

[43] Zhang, W. ; Zang, N. ; Jiang, Y. ; Chen, P. ; Wang, X. ; Zhang, X. Upregulation of Phosphodiesterase Type 5 in the Hyperplastic Prostate. Sci. Rep 2015, 5. 10.1038/srep17888. PMC4674741 26657792

[44] Rahrmann E. P., Collier L. S., Knutson T. P., Doyal M. E., Kuslak S. L., Green L. E., Malinowski R. L., Roethe L., Akagi K., Waknitz M., Huang W., Largaespada D. A., Marker P. C. (2009). Identification of PDE4D as a Proliferation Promoting
Factor in Prostate Cancer Using a Sleeping Beauty Transposon-Based
Somatic Mutagenesis Screen. Cancer Res..

[45] Andersson K. E., Uckert S., Stief C., Hedlund P. (2007). Phosphodiesterases
(PDEs) and PDE Inhibitors for Treatment of LUTS. Neurourol Urodyn.

[46] Sebastianelli, A. ; Spatafora, P. ; Morselli, S. ; Vignozzi, L. ; Serni, S. ; McVary, K. T. ; Kaplan, S. ; Gravas, S. ; Chapple, C. ; Gacci, M. Tadalafil Alone or in Combination with Tamsulosin for the Management for LUTS/BPH and ED. Curr Urol Rep 2020, 21 (12). 10.1007/s11934-020-01009-7. PMC7591403 33108544

