# Supplementary material for: Synthesis and Pharmacological Evaluation of 6,7-Dihydro‑3H‑Oxazolo[3,4‑a]Pyrazine-5,8-Dione Compounds as Inhibitors of Phosphodiesterases 4 and 5
Source: ACS Med Chem Lett. 2026 Jan 20;17(2):341–50. doi: 10.1021/acsmedchemlett.5c00525 (PMC12907935; doi:10.1021/acsmedchemlett.5c00525)
Supplement: Supplementary file 1 [file ml5c00525_si_001.pdf]

Supporting Information

Synthesis and Pharmacological Evaluation of 6,7-Dihydro-3H-Oxazolo[3,4-a]Pyrazine-5,8-Dione Compounds as Phosphodiesterases 4 and 5 Inhibitors

Débora Rocha Helfstein,[a,b] Marcio Fernando das Virgens,[b] Julio Alejandro Rojas Moscoso,[b] Tiago Zaminelli,[b] Fabiano Travanca. Toledo,[b] Jennifer Lima,[a] Larissa Ozols Medeiros, [b] Bianca Alves Marcello, [b] Vinícius Marques Soares, [b] Gabriela Reolon Passos,[a] Sarah Saraiva de Padua,[c] Matheus Eduardo Gonçalves Wolf,[c] Leonardo Martins Carneiro,[c] Gilberto De Nucci, [a] Artur Franz Keppler[c], Fabíola Zakia Mónica,[a]*

[a] Department of Pharmacology, Faculty of Medical Sciences, University of Campinas, Sao Paulo, Brazil.

[b] Biolab Sanus Pharmaceutical, São Paulo, Brazil.

[c] *Centro de Ciências Naturais e Humanas, Universidade Federal do ABC, Avenida dos Estados, 5001, Bloco A, Santo André 09210-580, São Paulo, Brazil*;

Materials and methods

Chemistry

General Information

All chemicals and solvents were used as received without further purification. Melting points were measured using a Büchi M-560 and are uncorrected. **^{1}H** and **^{13}C** NMR spectra were recorded on a Bruker 300 Fourier operating at 300 MHz for ^{1}H and 75 MHz for ^{13}C. Chemical shifts are reported in hertz (*Hz*). Deuterated dimethyl sulfoxide (DMSO-d_6) was used as the solvent. High-resolution mass spectrometry (HRMS) was carried out using a Waters Xevo G2-XS Tof instrument. Reaction progress was monitored by thin-layer chromatography (TLC) on silica gel 60 GF254 plates, using $CHCl_3$/MeOH (95:5) as the mobile phase.

General Procedure for 6,7-dihydro-3H-oxazolo[3,4-a]pyrazine-5,8dione Synthesis

In a 250 mL round-bottomed flask equipped with magnetic stirring, an addition funnel, and a drying tube containing CaCl2, (*E*)-methyl 2-((benzo[*d*][1,3]dioxol-5-ylmethylene)amino)-3-(1*H*-indol-3-yl)-3-oxopropanoate (**IV**) (5.1 g, 14 mmol) was added along with a solution of dry pyridine (5.6 mL, 70 mmol) in dry THF (90 mL). The mixture was stirred for 30 minutes. To this suspension, a solution of chloroacetyl chloride (1.6 mL, 19.6 mmol) in dry THF (20 mL) was added slowly over a period of about 2 hours. After the addition, the reaction mixture was stirred for an additional 4 hours at room temperature. At the end of this period, the corresponding primary amine (322 mmol) was added, and the mixture was stirred for 16 hours. After this time, THF was removed by distillation, and 100 mL of ethanol was added to the reaction flask. The mixture was stirred for an additional 2 hours at 0°C. After this, the solid was filtered under vacuum, and the precipitate was washed with ethanol. The obtained solid was dried in an oven at 85°C, yielding the desired product.

(**COMPOUND VI**)

3-(benzo[*d*][1,3]dioxol-5-yl)-1-(1*H*-indol-3-yl)-7-methyl-6,7-dihydro-3*H*-oxazolo[3,4-*a*]pyrazine-5,8-dione

Yield: 34%; yellow solid; P.F.: 279-281°C; ^{1}H RMN (300 MHz, DMSO-d_6): δ 11.86 (ls, 1H), 8.96 (s, 1H), 7.85 (d, *J*= 7.8 Hz, 1H), 7.46 (d, *J*= 8.0 Hz, 1H), 7.18-7.03 (m, 5H), 6.96 (d, *J*= 7.9 Hz, 1H), 6.04 (s, 2H), 4.17 (s, 2H), 2,92 (s, 3H); ^{13}C NMR (75 MHz, DMSO-d6): δ 158.5, 156.8, 148.4, 147.6, 147.5, 135.8, 131.1, 130.9, 125.2, 122.2, 121.4, 120.6, 120.5, 112.2, 108.4, 106.5, 104.8, 102.3, 101.5, 90.3, 52.9, 32.4; HRMS (EI) *m/z* calculated for $C_{22}H_{18}N_3O_5$ 404.1246 $[M+H]^+$, found 404,1676.

(COMPOUND VII)

3-(benzo[*d*][1,3]dioxol-5-yl)-1-(1*H*-indol-3-yl)-6,7-dihydro-3*H*-oxazolo[3,4-*a*]pyrazine-5,8-dione

Yield: 28%; yellow solid; P.F.: 268 – 273°C; ^{1}H NMR (300 MHz, DMSO-d_6): δ 11.84 (ls, 1H), 8.94 (d, *J*= 2.5 Hz, 1H), 7.87-7.83 (m, 2H), 7.47, (d, *J*= 8.0 Hz, 1H), 7.18-7.03 (m, 5H), 6.96 (d, *J*= 7.9 Hz, 1H), 6.04 (s, 2H), 4.08 (d, *J*= 17.2 Hz, 1H), 3.99 (d, *J*= 17.7 HZ, *J*= 1.7 Hz, 1H); ^{13}C NMR (75 MHz, DMSO-d6): δ 159.2, 158.3, 148.3, 147.7, 147.6, 135.8, 131.1, 131.0, 125.2, 122.3, 121.3, 120.7, 120.4, 112.3, 108.4, 106.5, 104.8, 102.3, 101.5, 90.5, 46.1; HRMS (EI) *m/z* calculated for $[C_{21}H_{15}N_3O_5]^+$: 389.1012, found: 390.1512 $[M+H]^+$.

(**COMPOUND VIII**)

3-(benzo[*d*][1,3]dioxol-5-yl)-7-(2-hydroxyethyl)-1-(1*H*-indol-3-yl)-6,7-dihydro-3*H*-oxazolo[3,4-*a*]pyrazine-5,8-dione

Yield: 45%; yellow solid; P.F.: 254-258°C; ^{1}H NMR (300 MHz, DMSO-d_6): δ 11.86 (ls, 1H), 8.95 (s, 1H), 7.85 (d, *J*= 7.8 Hz, 1H), 7.46 (d, *J*= 8.0 Hz, 1H), 7.18-7.03 (m, 5H), 6.97 (d, *J*= 8.0 Hz, 1H), 6.04 (s, 2H), 4.82 (t, *J*= 5.6, 1H), 4.30 (d, *J*= 17.6 Hz, 1H), 4.23 (d, *J*= 17.6, 1H), 3.65-3.60 (m, 2H), 3.49-3.45 (m, 2H); ^{13}C NMR (75 MHz, DMSO-d6): δ 159.0, 157.2, 148.8, 148.1 (2C), 136.3, 131.5, 131.3, 125.7, 122.7, 121.8, 121.1, 120.9, 112.7, 108.8, 107.0, 105.3, 102.7, 101.9, 90.8, 58.8, 52.9, 48.2; HRMS (EI) *m/z* calculated for $C_{23}H_{19}N_3O_6$: $[433.1274]^+$ found: 433,1683 $[M+H]^+$.

(**COMPOUND IX**)

3-(benzo[*d*][1,3]dioxol-5yl)-7-((*S*)-1-hydroxypropan-2-yl)-1-(1*H*-indol-3-yl)-6,7-dihydro-3*H*-oxazolo[3,4-*a*]pyrazine-5,8-dione

Yield: 21%; yellow solid. P.F.: 221-223 °C; ^{1}H NMR (300 MHz, DMSO-d_6): δ 11.86 (ls, 1H), 8.97 (d, *J*= 2.9 Hz, 1H), 7.87 (d, *J*= 8.0 Hz, 1H), 7.47 (d, *J*= 8.0 Hz, 1H), 7.18–7.04 (m, 5H), 6.97 (dd, *J*= 6.7 Hz, *J*= 1.7 Hz, 1H), 6.04 (s, 2H), 4.85 (t, *J*= 5.8 Hz, 1H), 4.67–

4.60 (m, 1H), 4.11 (d, *J*= 17.5 Hz, 1H), 4.04 (d, *J*= 17.5 Hz, 1H), 3.59-3.40 (m, 2H), 1.07 (d, *J*= 6.9 Hz, 3H); ^{13}C NMR (75 MHz, DMSO-d_6) δ 159.1, 157.1, 148.3, 147.7, 147.6, 135.8, 131.1, 130.9, 125.2, 122.2, 121.4, 120.6, 120.4, 112.2, 108.3, 106.5, 105.2, 102.4, 101.4, 90.2, 61.4, 49.4, 46.1, 13.2; HRMS (EI) *m/*z calculated for $[C_{24}H_{21}N_3O_6]^+$: 447.1430, found: 449.2005 $[M+Na]^+$.

(**COMPOUND X**)

3-(benzo[*d*][1,3]dioxol-5yl)-7-((*R*)-1-hydroxypropan-2-yl)-1-(1*H*-indol-3-yl)-6,7-dihydro-3*H*-oxazolo[3,4-*a*]pyrazine-5,8-dione

Yield: 25%; yellow solid; P.F: 246 – 248°C; ^{1}H NMR (300 MHz, DMSO-d_6): δ 11.85 (ls, 1H), 8.96 (d, *J*= 2.1 Hz, 1H), 7.86 (d, *J*= 7.9 Hz, 1H), 7.46 (d, *J*= 8.0 Hz, 1H), 7.18–7.03 (m, 5H), 6.97 (dd, *J*= 6.6 Hz, *J*= 1.8 Hz 1H), 6.04 (s, 2H), 4.85 (t, *J*= 5.8 Hz, 1H), 4.69–4.57 (m, 1H), 4.11 (d, *J*= 17.5 Hz, 1H), 4.04 (d, *J*= 17.5 Hz, 1H), 3.59–3.40 (m, 2H), 1.07 (d, *J*= 7.0 Hz, 3 H); ^{13}C NMR (75 MHz, DMSO-d6): δ 159.1, 157.1, 148.4, 147.7, 147.6, 135.8, 131.2, 130.9, 125.3, 122.2, 121.4, 120.6, 120.5, 112.3, 108.4, 106.6, 105.2, 102.4, 101.5, 90.2, 61.4, 49.4, 46.1, 13.2; HRMS (EI) *m/*z calculated for $[C_{24}H_{21}N_3O_6]^+$: 447.1430, found 4448.1992 $[M+H]^+$.

Biological methods

In vitro phosphodiesterases inhibition

Percentage of inhibition of phosphodiesterase subtype PDE4 family enzymes (PDE4A1A, PDE4B1, PDE4B2, PDE4C1, PDE4D2 and PDE4D3) was determined using Assay Kits from BPS Bioscience Inc., United States (Cat 60340, 79558, 60343, 60345 and 60346, respectively). Procedures were performed according to manufacture's instructions procedures. Compound are screened at 1 µM in triplicate. The assay was based on the binding of a fluorescent nucleotide monophosphate generated by each PDE subtype to the binding agent. The fluorescently labeled cAMP were incubated with a sample containing the enzyme for 1 hour. Then the binding agent was added to the reaction mix to produce a change in fluorescent polarization that can then be measured using a multimode microplate reader (Victor Nivo, Perkin Elmer, US) with excitation wavelength 485 nm and emission wavelength 528 nm. The control inhibitor used for this assays was Rolipram (Cat# R640040, TRC Inc) 1 µM.

PDE5 enzymatic activity was determined using a PDE5 assay kit (BPS Bioscience Inc., United States Cat 60350). Compound are screened at 1 µM in triplicate. The assay was based on the binding of a fluorescent nucleotide monophosphate generated by PDE5 to the binding agent. The fluorescently labeled cGMP were incubated with a sample containing the enzyme for 1 hour. Then the binding agent was added to the reaction mix

to produce a change in fluorescent polarization that can then be measured using a multimode microplate reader (Victor Nivo, Perkin Elmer, US) with excitation wavelength 470 nm and emission wavelength 528 nm. The control inhibitor used for this assays was Tadalafil (Cat# Y0001417, EDQM) 1 μM.

Evaluation of the effects of COMPOUND VIII on the activity of the human phosphodiesterase family enzymes was screened at 10 μM in duplicate, according the report number 100013463, carried out by Cerep Services, France.

Functional Assays in rat prostate tissue

Procedures with male Wistar Rats, 8 to 10 weeks old was approved by the Institutional Committee for Ethics in Animal Research/Nuclear Energy Research Institute (protocol number 19/22).

After euthanasia, the rat ventral prostate was isolated and two strips of prostate were mounted in a myograph (DNT820MS Muscle Strip System) filled with warm Krebs-Henseleit solution (mM: NaCl, 117; $NaHCO_3$, 14.9; Dextrose, 5.5; KCl, 4.7; KH_2PO_4, 1.18; $MgSO_47H_2O$, 1.17 e CaCl2; H_2O, 1.6, pH 7.4) and continuously bubbled with mixture of O_2: CO_2 (95:5 %) at 37 ºC. A basal tension of 5 mN was applied during 45 minutes equilibration period. After this period, the tissue was pre-contracted with phenylephrine (10 μm) and a curve-concentration response (100 pM a 100 μM) with COMPOUND VIII and Tadalafil was performed. Another two relaxation CCR was made, in the presence and abscence of COMPOUND VIII (3 μM), with SNP and Isoproterenol. To compare the results with Isoproterenol, a CCR with presence and absence of Roflumilast (110 μM), a PDE4 inhibitor was performed as well. In addition, the final CCR was a contraction curve with Phenylephrine, in the presence and in abscence of COMPOUND VIII (3 μM). The isometric force was recorded using PowerLab 400TM Data Aquisition System (Software Chart, version 6.0, AD Instrument, Milford, MA, United States).

Cells Proliferation assays

The human normal prostatic epithelial cell line (RWPE-1) was supplied by by Rio de Janeiro Cells Bank (BCRJ, Brazil). The cells were cultured in RPMI 1640 medium (product no. 31800022, Gibco, USA), supplemented with 10% fetal bovine serum (product no. 12657-029, Gibco, USA) and 1% antibiotic (penicillin-streptomycin,

product no. 15140-122, Gibco). The cells were incubated in a humidified 5% CO_2 atmosphere at 37 °C and were used between passages 16-22. The human benign prostatic hyperplasia cell line (BPH-1) was purchased from Merck (MA, USA) and the myofibroblast cell line from prostate stroma (WPMY) were supplied by ATTCC (USA), both were cultured as described for RWPE-1. The cells were used between passages 16-22.

Early passage BPH-1, RWPE-1 and WPMY were seeded ata density of 10,000 cells/well in 96-well plates in quadriplicates in 200µl RPMI 1640 (Gibco) culture médium containing 10% bovine fetal sérum and 1% penicillin/streptomycin. After adhesion (2 hours), cells were treated with COMPOUND VIII (300 nM, 1 µM and 3 µM), Tadalafil (300 nM, 1 µM, 3 µM and 10 µM), Rolipram (300 nM, 1 µM, 3 µM and 10 µM), and with the vehicle (PBS and DMSO 0,1%). Cell numbers were determined after 72 h of incubation and was assessed using a Cyquant™ NF kit (Thermo Fisher Scientific Inc.Ireland), according to the manufacturer's instructions and the fluorescence was measured by a multimode microplate reader (Victor Nivo, Perkin Elmer, US). The assay was conducted on three separate occasions. The data were expressed as percentages relative to the vehicle (DMSO 0,1%) which was considered 100%. Statistical significance vs controls was determined by One-way ANOVA followed by the Bonferroni multiple comparisons test.

Computational Methods

All computational calculations were performed using AutoDock Vina as implemented in the MolAr software suite (Molecular Architecture, version 1.13.1). The crystallographic structure of PDE5A was obtained from the Protein Data Bank (PDB ID: 1UDU). Protein preparation included protonation at pH 7.4 using the PDB2PQR server, simulating physiological conditions. During this process, crystallographic water molecules, metal ions, and the co-crystallized ligand were removed. The AMBER force field with the internal naming scheme was applied. To avoid introducing bias during structure preparation, the options "Ensure that new atoms are not rebuilt too close to existing atoms" and "Optimize the hydrogen bonding network" were deliberately disabled.

Molecular Docking

Compounds **VI–X** were docked into the cGMP-binding site of individual PDE5A monomers derived from the 1UDU structure using AutoDock Vina within MolAr. Ligands were prepared using AutoDock Tools, and docking calculations were performed with flexible ligands and a rigid receptor. The docking grid was defined based on the binding site of the reference inhibitor tadalafil, with the following parameters: center coordinates (X = 2.634, Y = 15.918, Z = 78.499), grid dimensions (X = 6 Å, Y = 10 Å, Z = 12 Å), and exhaustiveness set to 50. The lowest-energy binding poses were selected for interaction analysis. Two-dimensional interaction maps were generated using Discovery Studio 2025 to identify key ligand–protein contacts.

Redocking and Validation

To evaluate the robustness of the docking protocol, tadalafil was first redocked into the PDE5A catalytic site. The resulting pose closely reproduced the experimental crystallographic orientation, with an RMSD of 0.050 Å and a predicted binding affinity of −12.2 $kcal\cdot mol^{-1}$, supporting the reliability of the docking setup. Using the same parameters, compounds IX and X were subsequently redocked to further probe the interactions of their hydroxyethyl substituents within the H-loop region. In both cases, the highest-ranked poses converged back to their corresponding low-energy conformers, indicating a consistent relaxation toward preferred rotameric states. This behavior reinforces the notion that steric constraints surrounding the N7–C2 σ bond play a central role in dictating ligand orientation and conformational selection within the PDE5A binding pocket.

Visualization and ligand-receptor interaction analysis

Molecular visualization and analysis of ligand–protein interactions were performed using PyMOL. Docking output files were separated, and the lowest-energy poses were selected for detailed examination. Hydrogen-bond interactions and contacts involving the H-loop were explicitly highlighted to facilitate interpretation of ligand recognition and stabilization within the PDE5A catalytic site.

Conformational Analysis Methodology

To reduce computational complexity and focus on the core interactions, the chemical models were simplified by replacing the bulky benzo[1,3]dioxole and indole moieties with hydrogen atoms. These truncated analogues are herein designated as VII', IX', and X'. An initial exploration of the conformational space for these compounds was performed using the CREST (Conformer-Rotamer Ensemble Sampling Tool) program [6]. The resulting conformers were subsequently subjected to geometry optimization at the B3LYP/6-31G level of theory using the ORCA 5.0.4 software package [7]. Vibrational frequency calculations were performed to verify the stationary points, ensuring that all optimized geometries corresponded to true minima on the potential energy surface (i.e., absence of imaginary frequencies). Finally, to select the most representative structures, a clustering analysis based on Root Mean Square Deviation (RMSD) was conducted using in-house Python scripts utilizing the scikit-learn [8] and RDKit [9] libraries. This protocol resulted in the selection of three distinct geometries for each simplified compound.

References:

(1) https://doi.org/10.1002/pro.3280

(2) Eberhardt, J., Santos-Martins, D., Tillack, A.F., Forli, S.). AutoDock Vina 1.2.0: New Docking Methods, Expanded Force Field, and Python Bindings **2021**. Journal of Chemical Information and Modeling.

(3) Trott, O., & Olson, A. J.. AutoDock Vina: improving the speed and accuracy of docking with a new scoring function, efficient optimization, and multithreading. Journal of computational chemistry **2010**, 31(2), 455-461.

(4) BIOVIA, Dassault Systèmes, BIOVIA Workbook, Release 2020; BIOVIA Pipeline Pilot, Release 2020, San Diego: Dassault Systèmes, **2025**.

(5) The PyMOL Molecular Graphics System, Version 3.1 Schrödinger, LLC.

(6) Pracht, P.; Bohle, F.; Grimme, S. Automated exploration of the low-energy chemical space with fast quantum chemical methods (CREST) **2020**. Phys. Chem. Chem. Phys. 22, 7169–7192.

(7) Neese, F. Software update: the ORCA program system, version 4.0. WIREs Comput. Mol. Sci. 2018, 8, e1327.

(8) Pedregosa, F.; Varoquaux, G.; Gramfort, A.; et al. Scikit-learn: Machine Learning in Python. *J. Mach. Learn. Res.* **2011**, *12*, 2825–2830.

(9) Landrum, G. *RDKit: Open-source cheminformatics*. **2016**. Available at: http://www.rdkit.org.

Supplemental Figures

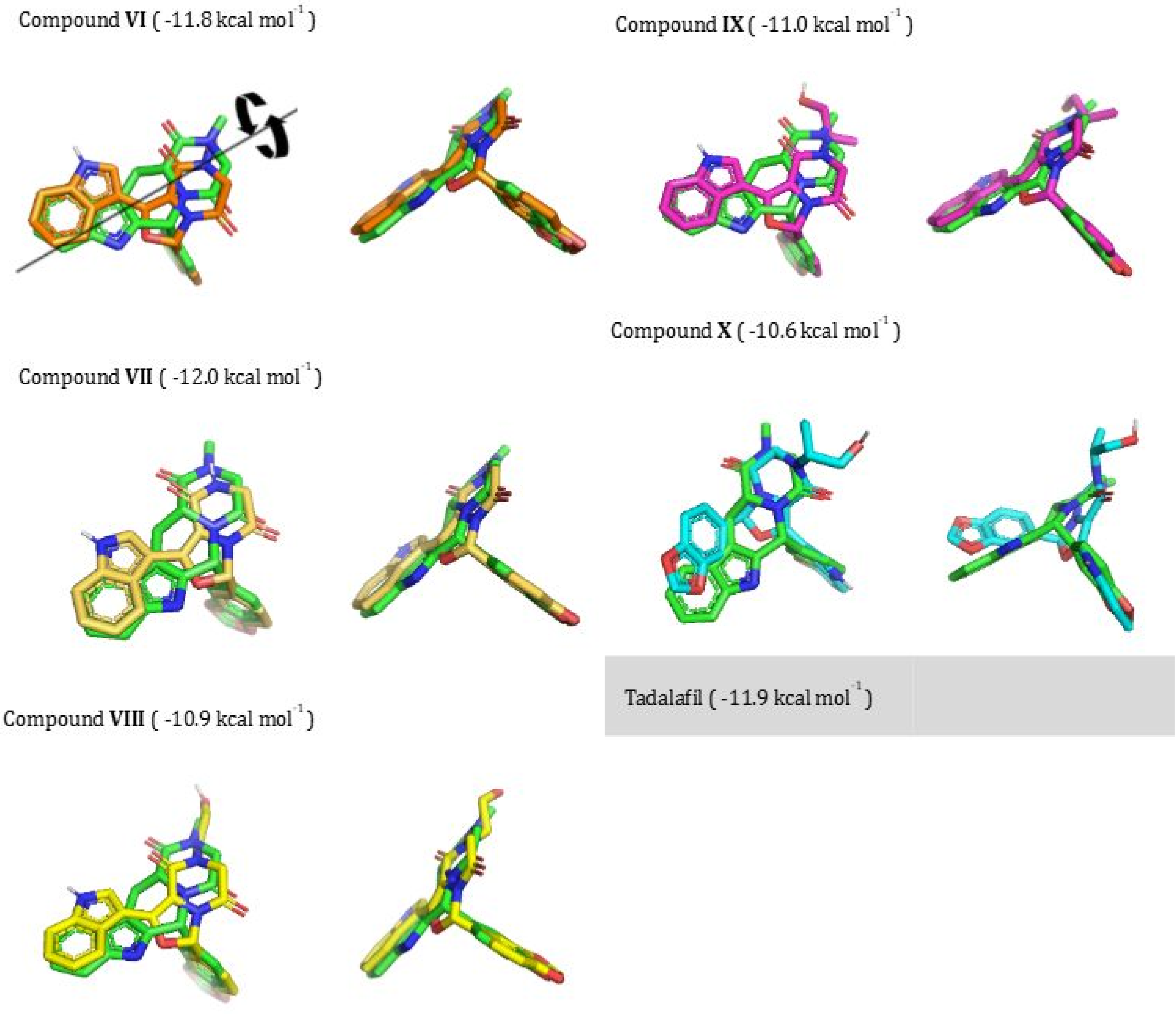

Figure S1. Comparison of the best docking poses obtained for **VI-X** and Tadalafil. Each panel presents a superposition of each compound with Tadalafil redocked pose. In parenthesis, the predicted binding affinity.

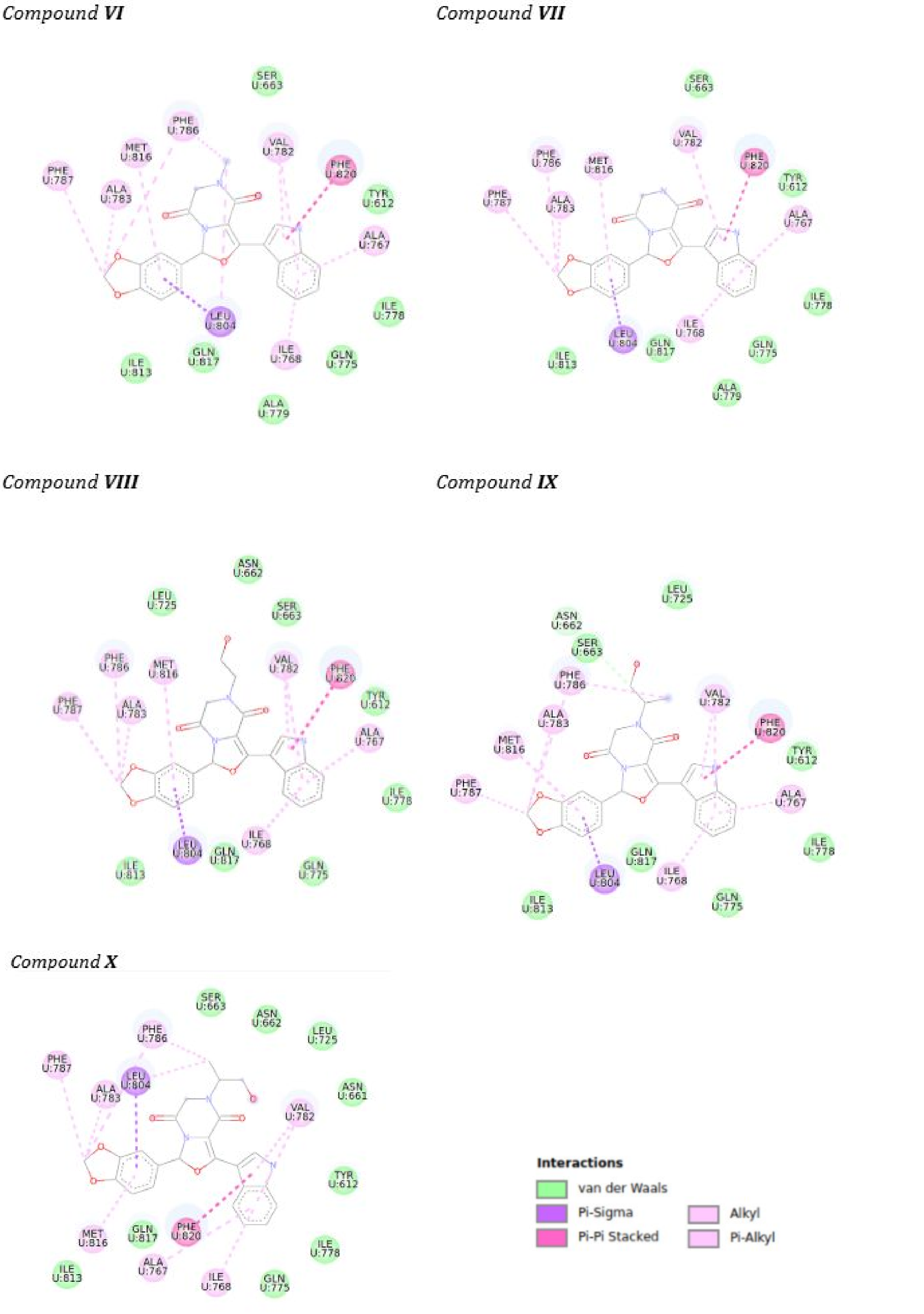

Figure S2: The Two-Dimensional interactions diagram for **VI-X**. All the chemical structures represent the best-ranked docking poses of each compound. Different colors identify the interactions with residues at the PDE5A catalytic pocket.

Compound **VIII**	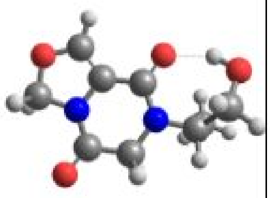	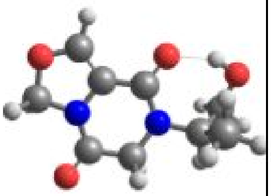	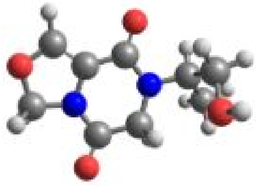
	0	$1{\cdot}10^{-5}$	3.36
Compound **IX**	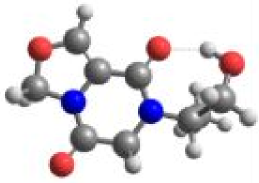	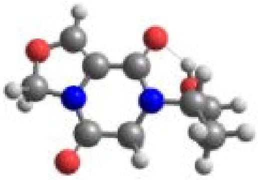	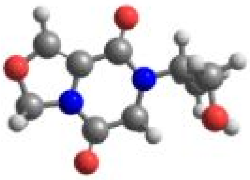
	0	1.13	3.72
Compound **X**	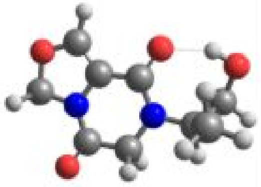	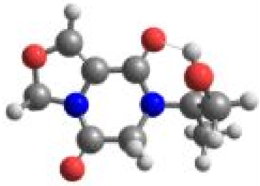	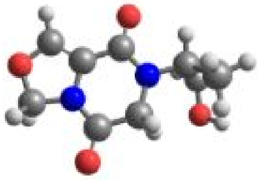
	0	1.13	3.72

Figure S3:. Three representative geometries for each simplified compound and their relative energies (kcal mol-1) with reference to minimum energy conformer (the first in each line).

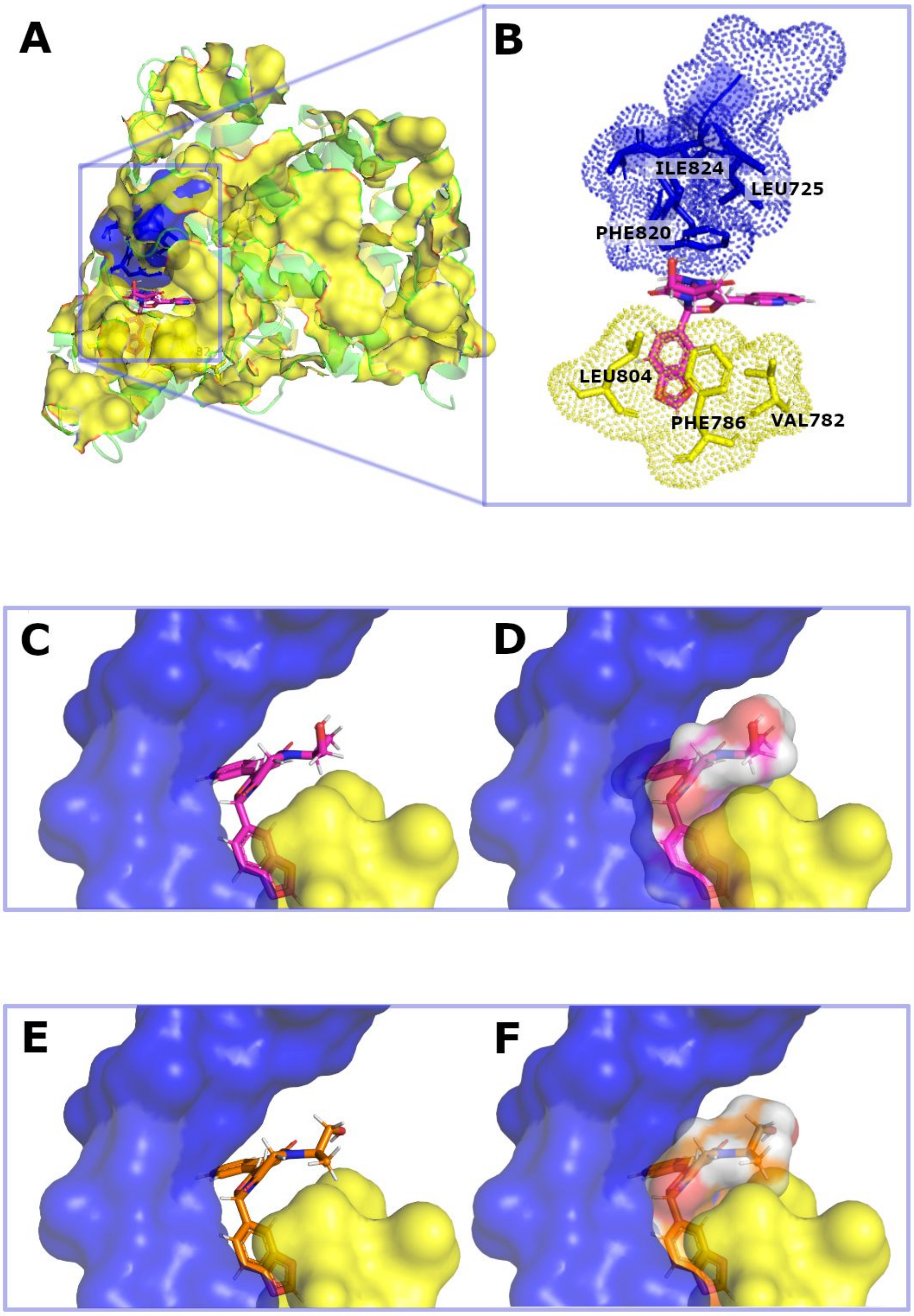

Figure S4: Best-ranked docking poses of compounds **IX** and **X** within the PDE5A cGMP-binding site. The yellow and blue surfaces depict the hydrophobic map. The protein tertiary structure is shown as a green cartoon with 60% transparency. **A.** Overview PDE5A with compound **IX** depicted as magenta sticks. **B.** Dotted surface representation of the cleft herein referred to as the hydroxyethyl-accommodating subpocket. **C**, **D**, **E** and **F**. Lateral views of this subpocket, highlighting the spatial orientation of the hydroxyethyl substituents of compounds **IX** (magenta) and **X** (orange), shown with and without a transparent surface to illustrate their corresponding electron density distribution.

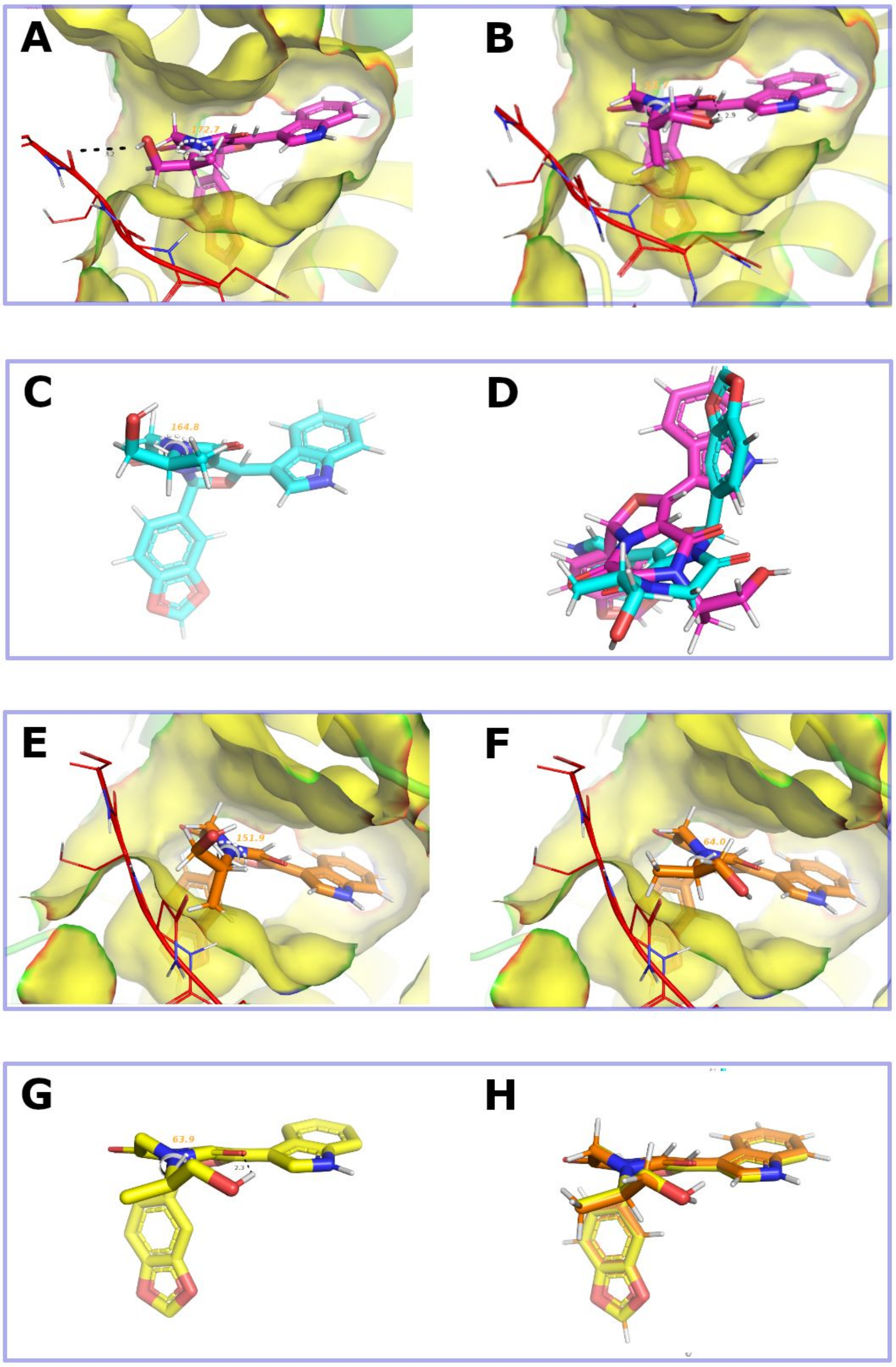
A
B
2.9
C
164.8
D
E
151.9
F
64.0
G
63.9
2.3
H

I

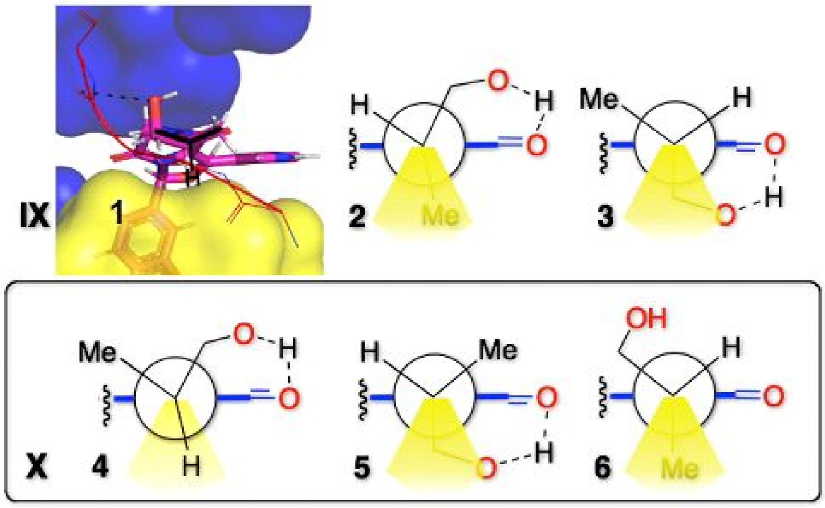

Figure S5: **A.** Best-ranking docking pose of **IX** (magenta sticks) - Conformer **1**. **B.** Controlled rotation about the N7-C2 bond, generating the Conformer **2**, that was used as input structure for the REDOCKING experiment. **C.** Best-ranking REDOCKING pose of **IX** (blue sticks), which relaxed back to conformer **1** and returned back with almost the same N7-C2 diedral angle (orange label). **D** Superposition of the best-ranking poses of docking (**IX** as magenta sticks) and redocking (**IX** as blue sticks) experiments. E-H. the same for X - best-ranking poses of docking (**X** as yellow sticks) and redocking (**IX** as orange sticks), **I.** conformational chart of N7-C2 bonds of **IX** and **X,** to facilitate the three-dimensional visualization.

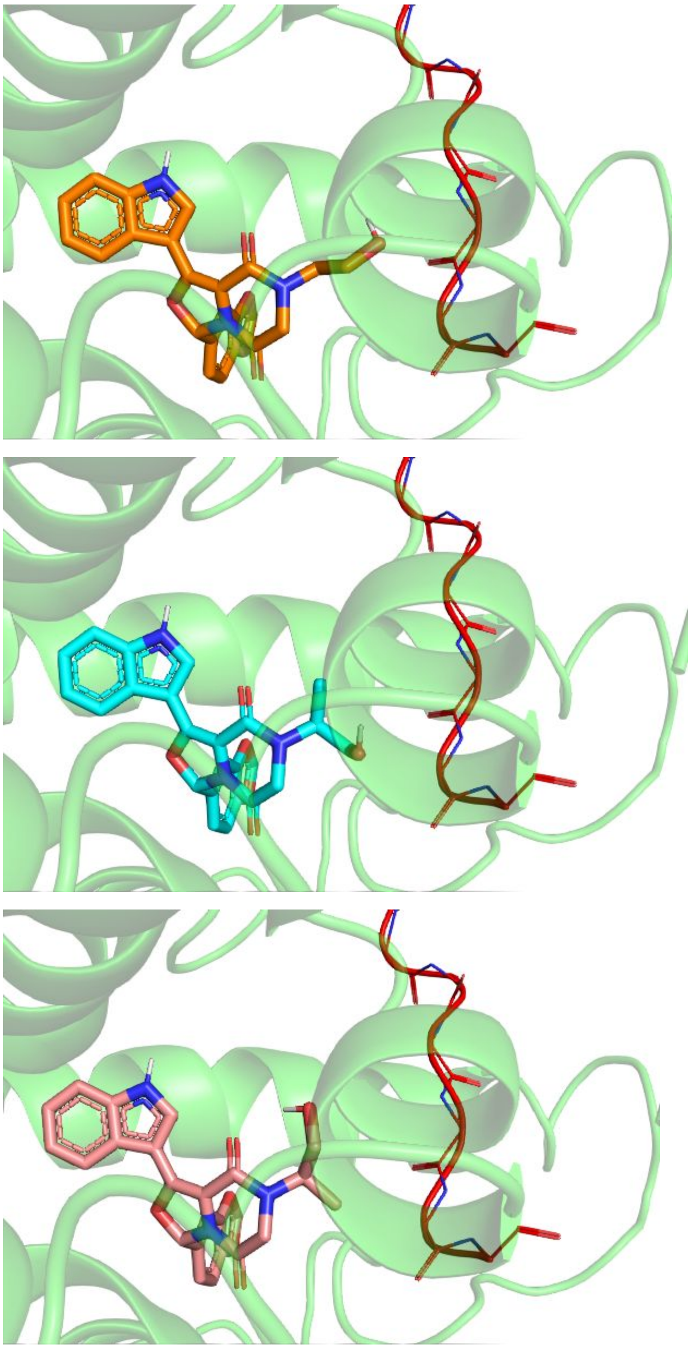

Figure S6: Crystal structure of PDE5A with the best docking poses obtained for ***VII, IX and X****, in a close-up view of the hydroxyethyl ligand pointed to the H-loop (red ribbon). Compounds* ***VIII, IX and X*** *are represented by the three-dimensional structures in orange, blue and salmon, respectively.*
